# Acarbose redirects gut microbiome utilization of dietary carbohydrates to suppress anaphylaxis in mice

**DOI:** 10.1038/s41564-026-02350-2

**Published:** 2026-05-12

**Authors:** Kyosuke Yakabe, Yukinobu Inoue, Yuki Yanagisawa, Shungo Imai, Shunnosuke Suwa, Masahiro Ando, Yuqing Wu, Rina Kurokawa, Tanakorn Srirat, Takeshi Haneda, Tsuyoshi Miki, Masahiro Ito, Akiyoshi Hirayama, Yosuke Kurashima, Shinji Fukuda, Koji Hase, Wataru Suda, Haruko Takeyama, Satoko Hori, Yun-Gi Kim

**Affiliations:** 1 Research Center for Drug Discovery, Faculty of Pharmacy and Graduate School of Pharmaceutical Sciences, Keio University, Tokyo, Japan; 2 Department of Microbiology, School of Pharmacy, Kitasato University, Tokyo, Japan; 3 Division of Drug Informatics, Faculty of Pharmacy and Graduate School of Pharmaceutical Sciences, Keio University, Tokyo, Japan; 4 Graduate School of Advanced Science and Engineering, Waseda University, Tokyo, Japan; 5 Research Organization for Nano and Life Innovation, Waseda University, Tokyo, Japan; 6 Department of Innovative Medicine, Graduate School of Medicine, Chiba University, Chiba, Japan; 7 Institute for Advanced Academic Research and Research Institute of Disaster Medicine, Chiba University, Chiba, Japan; 8 Chiba University, Synergy Institute for Futuristic Mucosal Vaccine Research and Development (cSIMVa), Chiba, Japan; 9 Department of Medicine, School of Medicine, Chiba University–University of California San Diego Center for Mucosal Immunology, Allergy and Vaccine (CU-UCSD cMAV), San Diego, CA USA; 10 Laboratory for Symbiotic Microbiome Sciences, RIKEN Center for Integrative Medical Sciences, Kanagawa, Japan; 11 Institute for Advanced Biosciences, Keio University, Yamagata, Japan; 12 Transborder Medical Research Center, University of Tsukuba, Ibaraki, Japan; 13 Gut Environmental Design Group, Kanagawa Institute of Industrial Science and Technology, Kanagawa, Japan; 14 Innovative Microbiome Therapy Research Center, Juntendo University Graduate School of Medicine, Tokyo, Japan; 15 Division of Biochemistry, Faculty of Pharmacy and Graduate School of Pharmaceutical Sciences, Keio University, Tokyo, Japan; 16 Research Management Division, The Institute of Fermentation Sciences (IFeS), Faculty of Food and Agricultural Sciences, Fukushima University, Fukushima, Japan; 17 Division of Mucosal Vaccine, International Vaccine Design Center, The Institute of Medical Science, The University of Tokyo (IMSUT), Tokyo, Japan; 18 Human Biology-Microbiome-Quantum Research Center (WPI-Bio2Q), Keio University, Tokyo, Japan; 19 Institute for Advanced Research of Biosystem Dynamics, Graduate School of Advanced Science and Engineering, Waseda Research Institute for Science and Engineering, Waseda University, Tokyo, Japan

**Keywords:** Molecular medicine, Allergy, Microbiome

## Abstract

Microbiota-accessible carbohydrates modulate host immunity by shaping gut microbial composition and metabolism. However, their role in modulating the microbiota to influence allergic responses is unclear. Here we show that a widely used antidiabetic agent, the α-glucosidase inhibitor acarbose, redirects dietary carbohydrate utilization by gut bacteria to suppress mast-cell-dependent anaphylaxis in mice, independently of adaptive immune responses. Enhanced carbohydrate availability promoted the proliferation of *Parabacteroides distasonis* in the mouse gut, leading to increased succinate abundance and intracellular NAD^+^ levels, and reduced reliance on b-type cytochrome-dependent anaerobic respiration. Direct administration of succinate suppressed systemic anaphylaxis and mast cell degranulation in vitro, implicating succinate as a key effector. A human cohort analysis revealed that patients treated with α-glucosidase inhibitors showed a lower incidence of anaphylaxis than untreated individuals. These findings uncover a previously unrecognized gut-microbiota-mediated pathway linking dietary carbohydrate metabolism to systemic immune regulation.

## Abstract (web summary)

Oral administration of an α-glucosidase inhibitor allows complex carbohydrates to reach the colon, where they promote succinate-producing bacteria that suppress anaphylaxis in mice.

## Main

Food allergy is triggered by degranulation of effector cells, such as mast cells and basophils, following binding of food antigens to IgE associated with the high-affinity IgE receptor (Fc epsilon receptor I alpha; FcεRIα). Clinical manifestations range from mild symptoms (cough, hives and diarrhoea) to severe systemic reactions such as anaphylaxis, an acute life-threatening condition whose incidence has increased in developed countries^1^. Genetic, epigenetic and environmental factors, including diet, pollutants and lifestyle, shape disease outcomes. In parallel, the gut microbiota has a central role in immune homeostasis and influences susceptibility to food allergy and anaphylaxis severity. Individuals with food allergies show altered microbial composition and metabolite profiles compared with healthy individuals^2,3^. Commensal microorganisms can protect against severe anaphylaxis by modulating ileal epithelial gene expression and inducing Retinoid-related orphan receptor (ROR) γt^+^ regulatory T (T_reg_) cells, thereby reducing antigen-specific IgE levels^4,5^. Consistently, germ-free (GF) mice show elevated serum IgE levels and enhanced anaphylaxis-induced hypothermia compared with specific pathogen-free (SPF) mice^6^. Thus, although the gut microbiota regulates anaphylaxis pathology, particularly allergen-specific IgE induction, the mechanisms by which it controls effector cell activation remain incompletely understood.

Gut microbiota composition and metabolic output are strongly influenced by diet^7,8^. Microbiota-accessible carbohydrates (MACs), including dietary fibre, are metabolized by gut bacteria^9,10^ and promote the production of metabolites such as short-chain fatty acids (SCFAs), which influence immune parameters including T_reg_ cell frequency and IgA responses^11,12^. Dietary fibres attenuate allergic responses through SCFA-dependent mechanisms^13,14^. However, MACs encompass diverse carbohydrate types, and individual microorganisms differ in their capacity to use these substrates and generate metabolites^9^. Consequently, their effects depend on both carbohydrate type and microbial context. Consistent with this concept, we previously showed that distinct carbohydrates can differentially or cooperatively shape microbial metabolism and host responses^15^.

Acarbose (Acr), an α-glucosidase inhibitor (α-GI) used to treat type 2 diabetes, inhibits α-amylase and α-glucosidase in the small intestine, thereby limiting carbohydrate digestion and suppressing postprandial glucose elevation^16^. As a result, undigested carbohydrates reach the colon^17^, where they serve as substrates for gut microorganisms and alter microbial composition in humans and mice^17–19^. Thus, Acr can be viewed as a pharmacological modulator of MACs. We hypothesized that Acr reshapes gut microbial metabolism by increasing carbohydrate availability in the colon, thereby modulating anaphylaxis.

To test this, we investigated whether pharmacological redirection of dietary carbohydrate metabolism by Acr alters microbial fermentation and metabolite production in a manner that suppresses systemic anaphylaxis. Specifically, we examined how Acr-driven metabolic changes translate into immune regulation. This study establishes a link between diet, microbial metabolism and allergic disease and suggests that microbiota-directed carbohydrate utilization may be leveraged to prevent severe allergic responses.

## Results

### Acr alleviates allergic diarrhoea and anaphylaxis

**Fig. 1:**
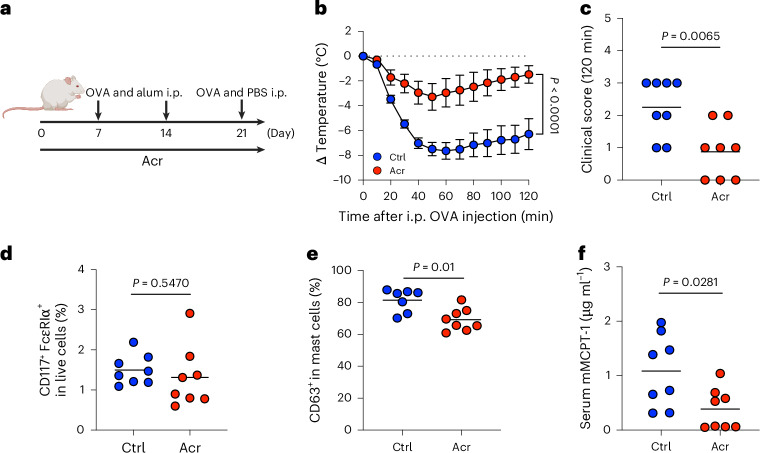
Acr alleviates systemic anaphylaxis by suppressing mast cell degranulation. **a**–**f**, Mice were sensitized with OVA and challenged intraperitoneally (i.p.) to induce systemic anaphylaxis. The body temperature was measured every 10 min for 120 min after the intraperitoneal injection (Ctrl, *n* = 8; Acr, *n* = 8). **a**, Experimental design. **b**, Change in body temperature. **c**, Clinical scores at 120 min after the challenge. **d**, Frequency of intraperitoneal mast cells in CD45^+^ live cells before the challenge. **e**, Frequency of CD63^+^ in mast cells 120 min after intraperitoneal challenge. **f**, Serum concentration of mMCPT-1 after intraperitoneal challenge. Each dot corresponds to an individual mouse or the mean ± s.e.m. The bar in the plots indicates the mean value. Statistical significance was assessed by two-way ANOVA with Sidak’s post hoc test (**b**) and two-sided Mann–Whitney *U* test (**c**, **d**, **e** and **f**). Illustration in **a** created in BioRender; Kim, Y. https://biorender.com/2l3k9jf (2026). Source data

**Extended Data Fig. 1:**
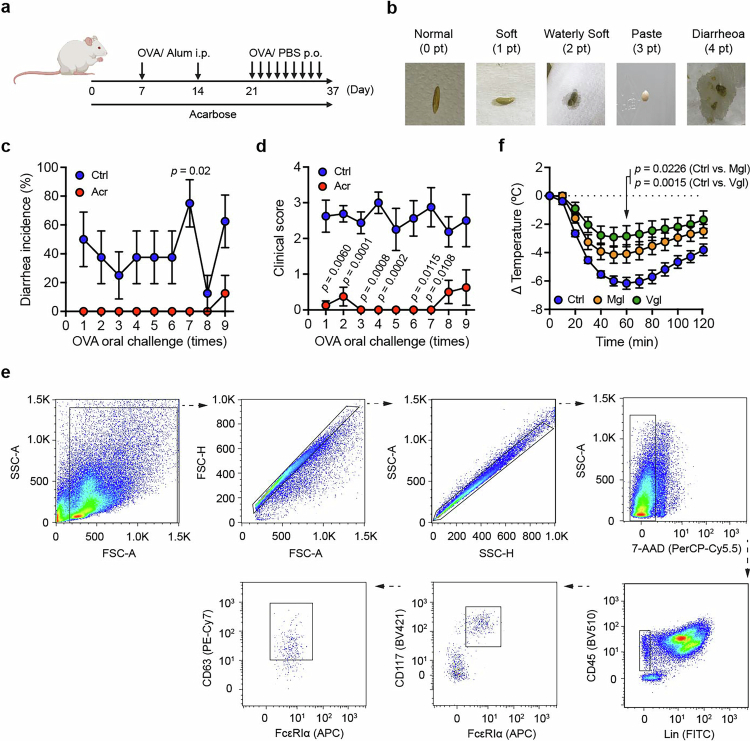
Acarbose suppresses OVA-induced allergic diarrhea. **a**-**d**, All mice were sensitized with OVA/alum and challenged with OVA by oral gavage nine times to induce food antigen–induced diarrhoea. **a**, Experimental design. **b**, Criteria used to assign clinical scores for diarrhoea. **c**, Incidence of diarrhoea. **d**, Clinical diarrhoea scores. Ctrl, n = 8; Acr, n = 8. **e**, Flow cytometry gating strategy for the identification of mast cells and degranulated mast cells in peritoneal lavage fluid. **f**, Mice were treated *ad libitum* with 0.125% miglitol (Mgl) or 0.0025% voglibose (Vgl) following the same experimental schedule shown in Fig. 1a. Core body temperature was measured every 10 min for 120 min after intraperitoneal OVA challenge. Ctrl, n = 14; Mgl, n = 15; Vgl, n = 15. Data are presented as mean ± SEM. Statistical significance was assessed by two-way ANOVA followed by Sidak’s post hoc test (**c**, **d**, and **f**). Ctrl, control; Acr, acarbose; OVA, ovalbumin; Mgl, miglitol; Vgl, voglibose. Illustration in **a** created in BioRender; Kim, Y. https://biorender.com/2l3k9jf (2026). Source data

To determine whether Acr alleviates allergic diarrhoea, mice were pretreated with Acr and challenged orally with ovalbumin (OVA) (Extended Data Fig. 1a), and clinical diarrhoea scores were evaluated (Extended Data Fig. 1b). Approximately 50% of control (Ctrl) mice developed diarrhoea, whereas Acr-treated mice rarely did (Extended Data Fig. 1c,d). We next assessed systemic anaphylaxis. Following sensitization, mice were challenged intraperitoneally with OVA (Fig. 1a). Ctrl mice showed a marked decrease in body temperature, whereas this reduction was attenuated in Acr-treated mice (Fig. 1b,c).

Because hypothermia reflects mast cell degranulation^20,21^, we evaluated mast cell responses. The frequency of mast cells in the peritoneal cavity was comparable between groups (Fig. 1d and Extended Data Fig. 1e), whereas the frequency of degranulated (CD63^+^) mast cells was significantly reduced in Acr-treated mice (Fig. 1e and Extended Data Fig. 1e). Consistently, serum mouse mast cell tryptase 1 (mMCPT-1) levels were lower in the Acr group (Fig. 1f). Finally, other GIs (miglitol (Mgl) and voglibose (Vgl)) similarly suppressed anaphylaxis (Extended Data Fig. 1f), indicating that this effect is a shared property of α-GIs.

### Acr suppresses mast cell degranulation independently of adaptive immunity

**Fig. 2:**
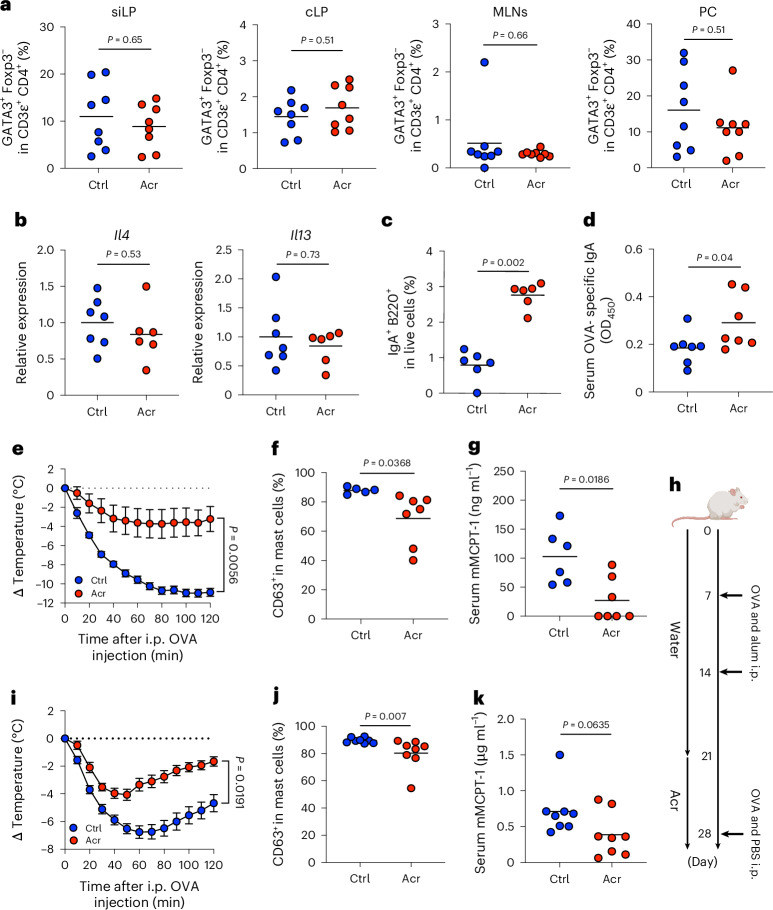
Acr suppresses the degranulation of mast cells and anaphylaxis independent of adaptive immune responses. **a**–**d**, Immune cell population, cytokine gene expression and antibody titres were analysed after sensitization. **a**, The frequency of GATA3^+^ Foxp3^−^ Th cells in the siLP, cLP, MLNs and PC (Ctrl, *n* = 8; Acr, *n* = 8). **b**, mRNA expression levels of *Il4* and *Il13* in MLNs (Ctrl, *n* = 7; Acr, *n* = 6). Relative expression was normalized relative to the Ctrl group = 1. **c**, Frequency of IgA^+^ B220^+^ cells in MLNs (Ctrl, *n* = 6; Acr, *n* = 6). **d**, Serum OVA-specific IgA levels (Ctrl, *n* = 7; Acr, *n* = 7). **e**–**g**, IgA KO mice were sensitized and intraperitoneally challenged with OVA. **e**, Change in body temperature (Ctrl, *n* = 8; Acr, *n* = 8). **f**, Frequency of CD63^+^ in mast cells in the PC (Ctrl, *n* = 5; Acr, *n* = 7). **g**, Frequency of CD63^+^ in mast cells (Ctrl, *n* = 6; Acr, *n* = 7). **h**–**k**, Mice were sensitized and intraperitoneally challenged with OVA to induce systemic anaphylaxis. Acr treatment started from 1 week after the 2nd sensitization (Ctrl, *n* = 8; Acr, *n* = 8). **h**, Experimental design. **i**, Change in body temperature. **j**, Frequency of CD63^+^ in mast cells. **k**, Concentration of serum mMCPT-1 after intraperitoneal OVA challenge. Each dot corresponds to an individual mouse or the mean ± s.e.m. The bar in the plots indicates the mean value. Statistical significance was assessed by two-way ANOVA with Sidak’s post hoc test (**e** and **i**) and two-sided Mann–Whitney *U* test (**a**, **b**, **c**, **d**, **f**, **g**, **j** and **k**). PC, peritoneal cavity. Illustration in **h** created in BioRender; Kim, Y. https://biorender.com/2l3k9jf (2026). Source data

**Extended Data Fig. 2:**
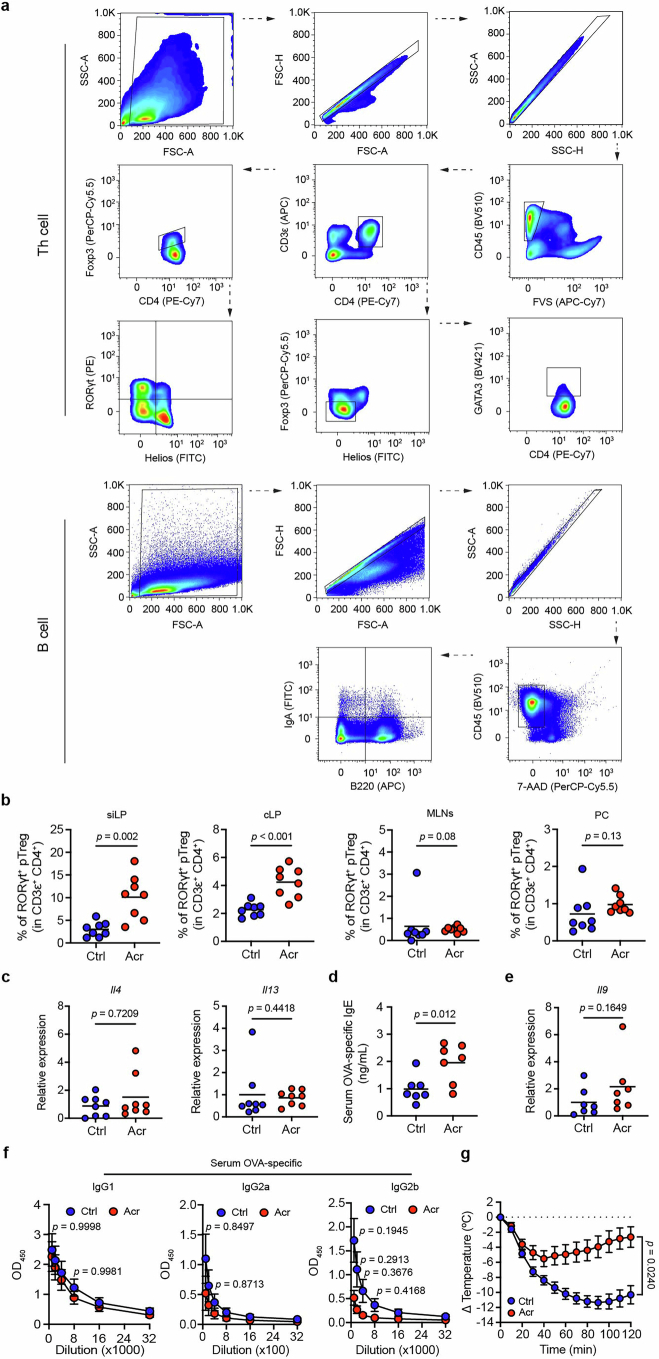
Changes in adaptive immune responses following Acr administration. **a**, Flow cytometry gating strategy for the identification of T helper (Th) cells and B cells. **b**, Frequency of RORγt^+^ peripheral regulatory T (pTreg; CD45^+^ CD3ε^+^ CD4^+^ Helios⁻ Foxp3^+^) cells in the small intestinal lamina propria (siLP), colonic lamina propria (cLP), mesenteric lymph nodes (MLNs), and peritoneal cavity (PC) (n = 8). **c**, mRNA expression levels of *Il4* and *Il13* in the small intestine (n = 8). **d**, Serum OVA-specific IgE concentrations measured on the day before challenge (day 21) (n = 7). **e**, mRNA expression levels of *Il9* in the small intestine (n = 7). **f**, Mean values of OVA-specific IgG subclasses (IgG1, IgG2a, and IgG2b) plotted across serum dilutions (n = 8). **g**, FcγRIIb knockout mice were sensitized and intraperitoneally challenged with OVA (n = 7), and changes in core body temperature were monitored. Each dot represents an individual mouse or the mean ± SEM, as indicated. Bars denote mean values. Relative expression levels were normalized to the Ctrl group (set to 1). Statistical significance was assessed by two-sided Mann–Whitney U test (**b**, **c**, **d**, and **e**) and by two-way ANOVA followed by Sidak’s post hoc test in (**f** and **g**). Ctrl, control; Acr, acarbose; OVA, ovalbumin; OD_450_, optical density at 450 nm. Source data

Allergic diseases are typically associated with type 2 immune responses and allergen-specific IgE^22^. Because Acr suppressed mast cell degranulation (Fig. 1), we examined whether it modulates adaptive immunity. Although RORγt^+^ T_reg_ cells were increased in the small intestinal and colonic lamina propria (siLP and cLP) of Acr-treated mice (Extended Data Fig. 2a,b), Th2 cell frequencies were unchanged compared with controls (Fig. 2a). Consistently, *Il4* and *Il13* expression levels in mesenteric lymph nodes (MLNs) and the small intestine were comparable between groups (Fig. 2b and Extended Data Fig. 2c). Despite this, serum OVA-specific IgE levels were higher in Acr-treated mice (Extended Data Fig. 2d). *Il9* expression in the small intestine was also unchanged (Extended Data Fig. 2e). We next examined FcγRIIb-mediated inhibitory signalling. Acr treatment did not alter OVA-specific IgG subclasses (Extended Data Fig. 2f), and its protective effect on body temperature was preserved in FcγRIIb-deficient mice (Extended Data Fig. 2g), indicating that this pathway is not involved. Because allergen-specific IgA can inhibit mast cell activation^23^, we assessed its contribution. Acr increased IgA^+^ B cells in MLNs and serum OVA-specific IgA levels (Fig. 2c,d and Extended Data Fig. 2a). However, Acr still suppressed hypothermia, mast cell degranulation and mMCPT-1 levels in IgA-deficient mice (Fig. 2e–g), indicating that IgA is not required. Finally, Acr treatment initiated after sensitization (day 21–28) still suppressed hypothermia, mast cell degranulation and mMCPT-1 levels following OVA challenge (Fig. 2h–k). Together, these results show that Acr suppresses mast cell degranulation and systemic anaphylaxis independently of adaptive immune responses.

### Acr suppresses anaphylaxis via the gut microbiota

**Fig. 3:**
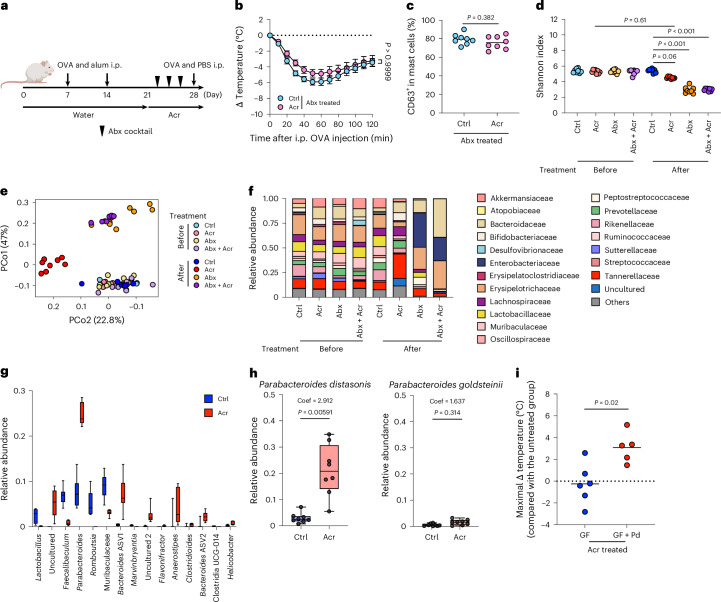
Acr suppresses anaphylaxis in a manner dependent on the gut microbiota. **a**–**c**, Mice were sensitized and intraperitoneally challenged with OVA. Mice were treated with Abx cocktail three times in 1 week after the 2nd sensitization. Faecal samples collected on days 21 and 28 (before the challenge) were used for 16S rRNA gene sequencing (*n* = 8). **a**, Experimental design. **b**, Change in body temperature. **c**, Frequency of CD63^+^ in mast cells in the PC. **d**–**g**, 16S rRNA gene sequencing was performed with the faecal samples collected before and after treatment (*n* = 8). **d**, α-diversity was evaluated using the Shannon index. **e**, β-diversity was analysed using principal coordinate analysis with weighted UniFrac distance. **f**, Relative abundance of each bacterial family. ‘Others’ includes the bacterial family whose sum is less than 25% in all groups. **g**, Relative abundance of significantly different bacterial genera (FDR < 0.05). The top 15 bacteria were selected based on the *q*-value. **h**, Box plots showing the relative abundances of *P. distasonis* and *P. goldsteinii* in the Ctrl (*n* = 8) and Acr (*n* = 8). Black points indicate individual samples. The MaAsLin3 model abundance coefficient (coef) and the corresponding *P* value (*P*) are shown in each panel. All box plots in **g** and **h** are Tukey style. The lower and upper hinges indicate the first and third quartiles. The upper whisker extends to the largest value within 1.5 × interquartile range from the hinge, and the lower whisker extends to the smallest value within 1.5 × interquartile range. Data beyond the whiskers are plotted individually. The horizontal line within each box indicates the median. **i**, Relative difference in the body temperature comparing the GF and *P. distasonis*-mono-associated GF treated with Acr at 50 min after the OVA challenge (GF; *n* = 6, GF + Pd; *n* = 5). Each dot corresponds to an individual mouse or the mean ± s.e.m. The bar in the plots indicates the mean value. Statistical significance was assessed by two-way ANOVA with Sidak’s post hoc test (**b**), two-sided Mann–Whitney *U* test (**c** and **i**) and one-way ANOVA with Dunn’s multiple-comparison post hoc test (**d**); MaAsLin3, with two-sided regression-based tests were used, and *P* values were adjusted for multiple comparisons using the Benjamini–Hochberg FDR method (**h**). Abx, antibiotic cocktail; GF + Pd, *P. distasonis*-mono-associated GF mice. Illustration in **a** created in BioRender; Kim, Y. https://biorender.com/2l3k9jf (2026). Source data

**Extended Data Fig. 3:**
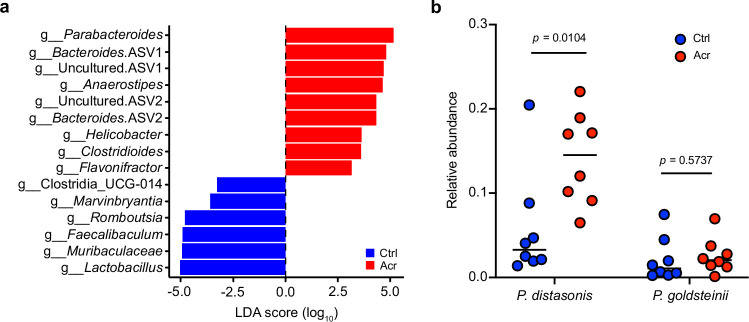
*P. distasonis* is the most abundant *Parabacteroides* species in acarbose-treated mice. **a**, LEfSe analysis performed using the microbiota dataset shown in Fig. 3f (day 21) (n = 8). Statistical significance was assessed using the Wilcoxon signed-rank test (*p* < 0.05), and the linear discriminant analysis (LDA) score threshold was set to >2.0. **b**, Relative abundance of *P. distasonis* and *P. goldsteinii* quantified by qPCR. Relative abundance was calculated by normalizing the gene expression level of each *Parabacteroides* species to total bacterial 16S rRNA gene expression. Statistical significance was determined using the two-sided Mann–Whitney U test. Ctrl, control; Acr, acarbose. Source data

Given the role of the gut microbiota in regulating anaphylaxis^4,5^, we tested whether Acr-mediated protection depends on microbial communities. Antibiotic (Abx) treatment abolished the protective effect of Acr on systemic anaphylaxis (Fig. 3a,b), and the frequency of degranulated mast cells became comparable between groups (Fig. 3c), indicating microbiota dependence. We next analysed microbial composition by 16S rRNA gene sequencing. Acr treatment modestly reduced α-diversity, whereas Abx markedly decreased it (Fig. 3d). Principal coordinate analysis of β-diversity showed that Acr-treated mice clustered separately from Ctrls, whereas Abx-treated groups clustered together (Fig. 3e). Acr treatment increased the relative abundance of Bacteroidaceae, Bifidobacteriaceae, Lachnospiraceae and Tannerellaceae and decreased Erysipelotrichaceae, Peptostreptococcaceae and Sutterellaceae (Fig. 3f). Linear discriminant analysis Effect Size (LEfSe) analysis identified *Parabacteroides*, *Bacteroides* and *Anaerostipes* as enriched taxa (Extended Data Fig. 3a), with *Parabacteroides* showing the highest relative abundance (Fig. 3g). Full-length 16S rRNA gene sequencing further identified *Parabacteroides distasonis* as the dominant species (Fig. 3h and Supplementary Table 1), which was confirmed by species-specific qPCR (Extended Data Fig. 3b). To assess causality, we colonized GF mice with *P. distasonis*. Acr treatment did not suppress anaphylaxis in GF mice, but markedly attenuated hypothermia following *P. distasonis* colonization (Fig. 3i), indicating that Acr exerts microbiota-dependent protection, at least in part via enrichment of *P. distasonis*.

### Succinate suppresses mast cell degranulation and anaphylaxis

**Fig. 4:**
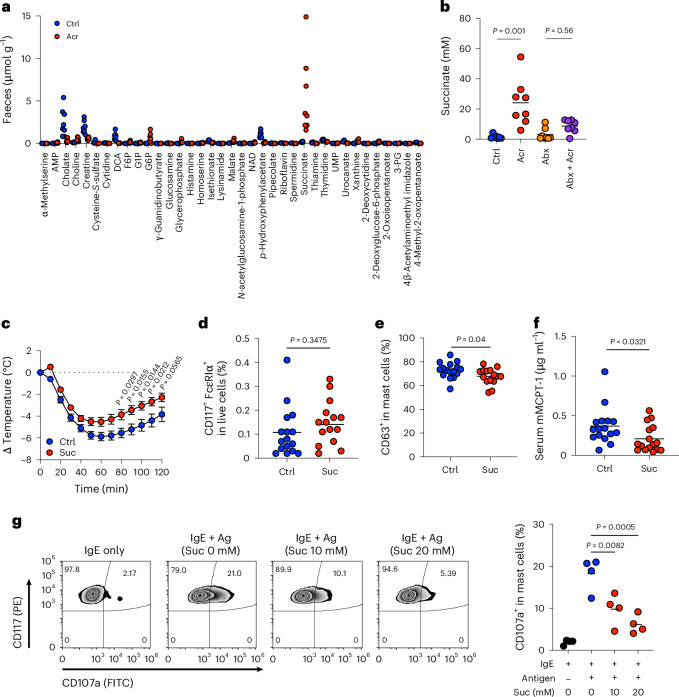
Succinate suppresses systemic anaphylaxis and mast cell degranulation. **a**, The concentration of faecal metabolites (two-sided Mann–Whitney *U* test; *P* < 0.001) was determined by CE-TOFMS (*n* = 8). **b**, The faecal levels of succinate were determined by GC–MS (Ctrl, *n* = 7; Acr, *n* = 8; Abx, *n* = 8; Abx + Acr, *n* = 8). **c**–**f**, Mice were sensitized and challenged with OVA intraperitoneally. Mice were administered 100 mM of succinate in drinking water for 1 week after the 2nd sensitization. **c**, Change in body temperature (Ctrl, *n* = 16; Suc, *n* = 16). **d**, Frequency of intraperitoneal mast cells in CD45^+^ cells (Ctrl, *n* = 16; Suc, *n* = 15). **e**, Frequency of CD63^+^ in mast cells after intraperitoneal OVA challenge (Ctrl, *n* = 16; Suc, *n* = 15). **f**, Serum concentration of mMCPT-1 after intraperitoneal OVA challenge (Ctrl, *n* = 16; Suc, *n* = 16). **g**, In vitro mast cell degranulation assay based on surface CD107a expression. Mast cells were pretreated with 10 mM or 20 mM Suc before antigen stimulation (*n* = 4 technical replicates). Each dot corresponds to an individual mouse, technical replicates or the mean ± s.e.m. The bar in the plots indicates the mean value. Statistical significance was assessed by one-way ANOVA with Dunn’s multiple post hoc comparisons test (**b** and **g**), two-way ANOVA with Sidak’s post hoc test (**c**) and two-sided Mann–Whitney *U* test (**a**, **d**, **e** and **f**). Suc, succinate. Source data

**Extended Data Fig. 4:**
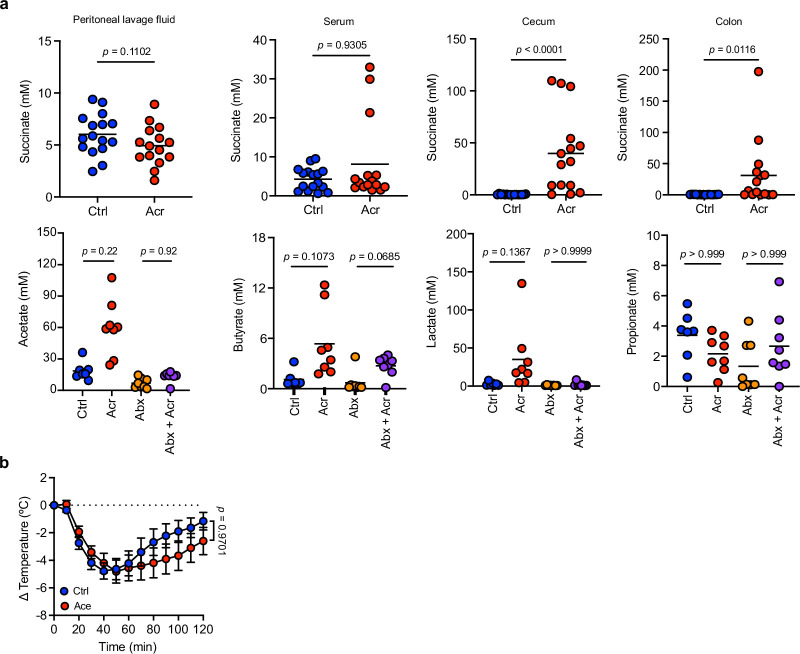
Acetate does not suppress systemic anaphylaxis. **a**, Succinate concentrations in peritoneal lavage fluid, serum, cecum, and colon (Ctrl; n = 16, Acr; n = 15), and fecal concentrations of acetate, butyrate, lactate, and propionate (Ctrl; n = 7, Acr, Abx, and Abx+Acr; n = 8), measured on the day before challenge (day 21). **b**, Mice were sensitized with OVA/alum and intraperitoneally challenged with OVA. Acetate (200 mM) was administered for 1 week after the second sensitization (Ctrl; n = 5, Ace; n = 8). Each dot represents an individual mouse, and bars indicate the mean ± SEM. Statistical significance was assessed using the two-sided Mann–Whitney U test (upper) and the Kruskal–Wallis test followed by post hoc multiple comparisons (lower) (**a**), and by two-way ANOVA followed by Sidak’s post hoc test in (**b**). Ctrl, control; Acr, acarbose; Ace, acetate; Abx, antibiotic cocktail. Source data

Gut microbial metabolites regulate host immune responses^11–14,24^. To identify metabolites associated with Acr treatment, we performed metabolomic analysis using capillary electrophoresis-time-of-flight mass spectrometry (CE-TOFMS). Among differentially abundant metabolites (two-sided Mann–Whitney *U* test; *P* < 0.001), succinate was significantly increased in the faeces of Acr-treated mice compared with controls (Fig. 4a). This increase was abolished by Abx treatment, indicating a microbiota-dependent origin (Fig. 4b). Consistently, succinate levels were elevated in the caecal and colonic lumen but not in serum or peritoneal fluid (Extended Data Fig. 4a). We next tested whether microbial metabolites suppress anaphylaxis. Oral administration of succinate, but not acetate, attenuated hypothermia following OVA challenge (Fig. 4c and Extended Data Fig. 4b). The frequency of mast cells was unchanged (Fig. 4d), whereas the proportion of degranulated (CD63^+^) mast cells and serum mMCPT-1 levels were significantly reduced (Fig. 4e,f). To assess direct effects, mast cells derived from peritoneal lavage were pretreated with succinate before antigen stimulation. Succinate significantly reduced the proportion of CD107a^+^ cells, indicating suppressed mast cell degranulation (Fig. 4g). Together, these data indicate that Acr increases microbiota-derived succinate, which suppresses mast cell degranulation and systemic anaphylaxis.

### *P. distasonis* mediates Acr-dependent suppression of anaphylaxis

**Fig. 5:**
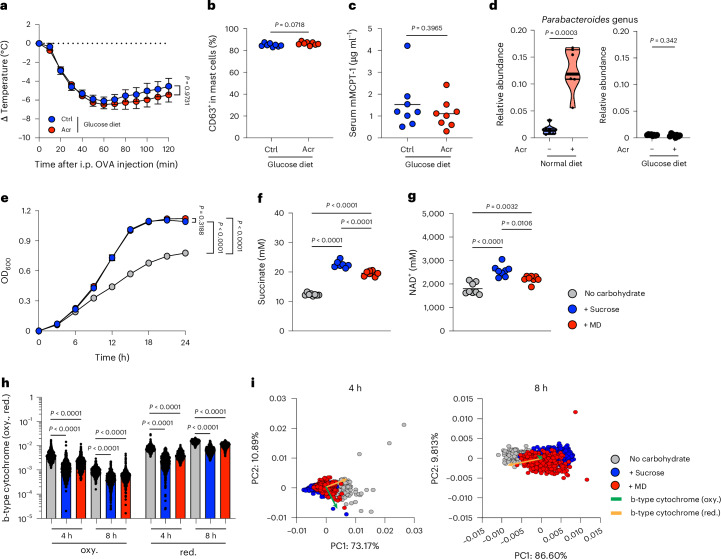
Carbohydrate-driven expansion and metabolic remodelling of *P. distasonis* underlie Acr-dependent suppression of anaphylaxis. **a**–**d**, Mice fed with a glucose diet were sensitized and challenged with OVA intraperitoneally (*n* = 8). **a**, Change in body temperature. **b**, Frequency of CD63^+^ in mast cells after intraperitoneal OVA challenge. **c**, Serum concentration of mMCPT-1 after intraperitoneal OVA challenge. **d**, Relative abundance of *Parabacteroides* was depicted based on the result of 16S rRNA gene amplicon sequencing. **e**–**g**, *P. distasonis* was cultured anaerobically in each modified GAM medium (without carbohydrates) containing sucrose or MD. **e**, The growth of *P. distasonis* was evaluated by measuring the OD_600_ every 3 h for 24 h (*n* = 5 technical replicates). **f**, Concentration of succinate in the culture supernatants of each *P. distasonis*-cultured medium at 24 h (*n* = 8 technical replicates). **g**, Intracellular NAD^+^ levels in *P. distasonis* after 12 h of culture (*n* = 8 technical replicates). **h**, Time-course analysis of reduced and oxidized b-type cytochrome levels in *P. distasonis* cultured with different carbohydrates, as assessed by Raman-MCR spectroscopy (*n* = 441 spectra). oxy., oxidized; red., reduced. **i**, PCA score plots based on Raman-MCR spectra of *P. distasonis* cultured under different carbohydrate conditions at 4 h and 8 h. Arrows indicate the factor loadings of reduced and oxidized b-type cytochrome components, with arrow lengths scaled to 1/40 of their loading values. Each dot corresponds to an individual mouse, bacterium or the mean ± s.e.m. The bar in the plots indicates the mean value. Statistical significance was assessed by two-way ANOVA with Sidak’s post hoc test (**a**), two-way ANOVA with uncorrected Fisher’s least significant difference (LSD) post hoc test (**e**), two-sided Mann–Whitney *U* test (**b**, **c** and **d**) and Tukey’s multiple-comparison test (**f**, **g** and **h**). Raman-MCR, Raman spectroscopy with multivariate curve resolution. Source data

**Extended Data Fig. 5:**
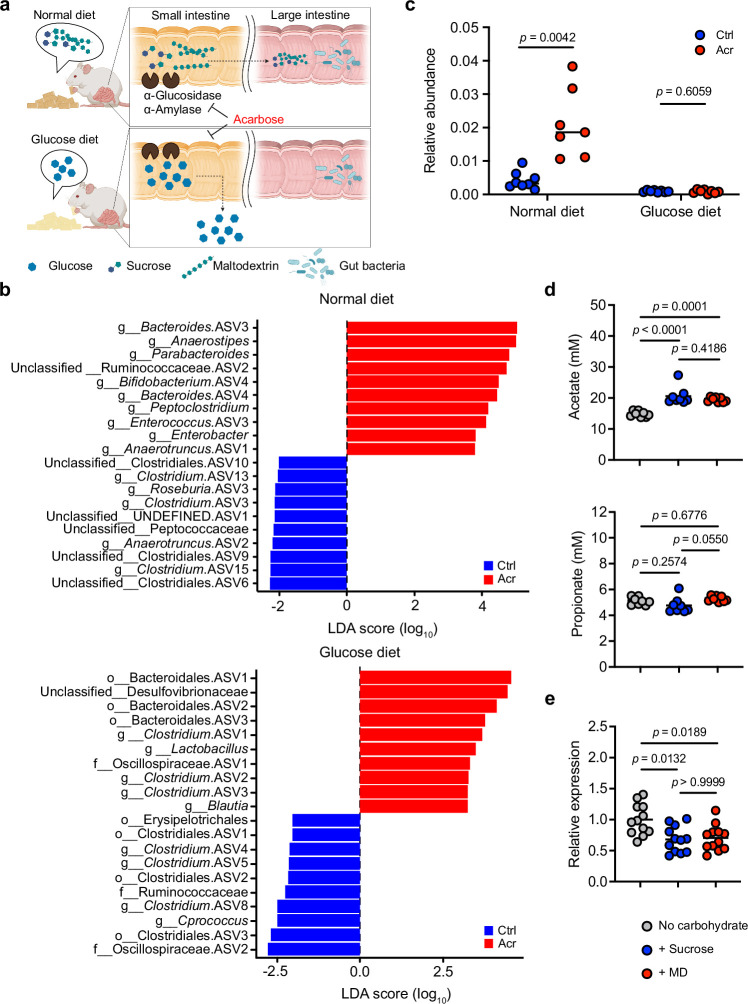
Digestible carbohydrates are essential for the expansion and metabolic remodeling of *P. distasonis*. **a**, Schematic illustration depicting the intestinal dynamics of dietary carbohydrates in mice. **b**, LEfSe analysis performed using the microbiota dataset shown in Fig. 5d (day 21) (Normal diet; Ctrl; n = 8, Acr; n = 7, Glucose diet; Ctrl; n = 8, Acr; n = 8). The upper panel shows comparisons among mice fed the normal diet (standard AIN-93G diet), and the lower panel shows comparisons among mice fed the glucose diet. Statistical significance was assessed using the Wilcoxon signed-rank test (*p* < 0.05), and the linear discriminant analysis (LDA) score threshold was set to >2.0. **c**, Relative abundance of *P. distasonis* in fecal samples from mice fed either the normal diet or the glucose diet, as determined by qPCR (Normal diet; Ctrl; n = 8, Acr; n = 7, Glucose diet; Ctrl; n = 8, Acr; n = 8). **d**, Concentrations of acetate and propionate in the culture supernatants of *P. distasonis* (No carbohydrate; n = 8, +Sucrose; n = 7, +MD; n = 8 technical replicates). **e**, Expression levels of the b-type cytochrome gene in *P. distasonis* after 4 h of culture, as determined by qPCR (n = 12 technical replicates). Each dot represents an individual mouse or an independent culture sample. Statistical significance was assessed using the two-sided Mann–Whitney U test (**c**) and the Kruskal–Wallis test followed by post hoc multiple comparisons (**d** and **e**). Ctrl, control; Acr, acarbose; MD, maltodextrin. Illustration in **a** created in BioRender; Kim, Y. https://biorender.com/z532k18 (2026). Source data

Acr increases the delivery of dietary carbohydrates normally digested in the small intestine to the colon through α-glucosidase inhibition^17–19,25^. To determine whether these carbohydrates are required for Acr-mediated protection, we used a glucose-based diet in which dietary digestible carbohydrates were replaced with D-glucose (Extended Data Fig. 5a and Supplementary Table 2). Under this condition, Acr failed to suppress systemic anaphylaxis (Fig. 5a), and both mast cell degranulation and serum mMCPT-1 levels were comparable between groups (Fig. 5b,c), indicating that dietary digestible carbohydrates are required for the protective effect of Acr. Consistent with this, Acr increased the relative abundance of *Parabacteroides* under a standard diet but not under the glucose diet (Fig. 5d and Extended Data Fig. 5b,c), suggesting that the availability of dietary digestible carbohydrates drives the expansion of this taxon. We next examined whether specific carbohydrates promote the growth and metabolic activity of *P. distasonis*. In vitro, both maltodextrin (MD) and sucrose enhanced bacterial growth and succinate production, with sucrose showing a stronger effect (Fig. 5e,f). Acetate also increased to a similar extent with both carbohydrates, indicating that acetate production is compatible with succinate production and not competitively suppressed (Extended Data Fig. 5d). Propionate showed a modest reduction with sucrose and no significant change with MD, suggesting limited flux towards this pathway (Extended Data Fig. 5d). Butyrate and lactate were barely detectable, indicating that *P. distasonis* does not meaningfully engage these pathways under the tested conditions^26,27^. Given that succinate production is linked to redox metabolism^28^, we measured intracellular nicotinamide adenine dinucleotide (NAD)^+^ levels to evaluate how carbohydrate availability alters the metabolic state of *P. distasonis*. Carbohydrate supplementation increased NAD^+^ levels, particularly under sucrose conditions (Fig. 5g), consistent with enhanced fermentative metabolism. We next assessed respiratory components associated with anaerobic metabolism. Raman spectroscopy revealed reduced levels of b-type cytochromes under carbohydrate-rich conditions, which was supported by decreased expression of b-type cytochrome genes (Fig. 5h and Extended Data Fig. 5e). Over time, reduced b-type cytochrome increased, whereas oxidized forms decreased, consistent with dynamic changes in redox state. Principal component analysis (PCA) further showed distinct metabolic states depending on carbohydrate availability (Fig. 5i), with clearer separation at later time points. Together, these findings indicate that dietary digestible carbohydrates promote the expansion of *Parabacteroides* and drive a coordinated metabolic shift towards succinate-producing fermentation, accompanied by reduced reliance on anaerobic respiratory pathways.

### α-GIs are associated with reduced anaphylaxis in diabetic patients

**Extended Data Fig. 6:**
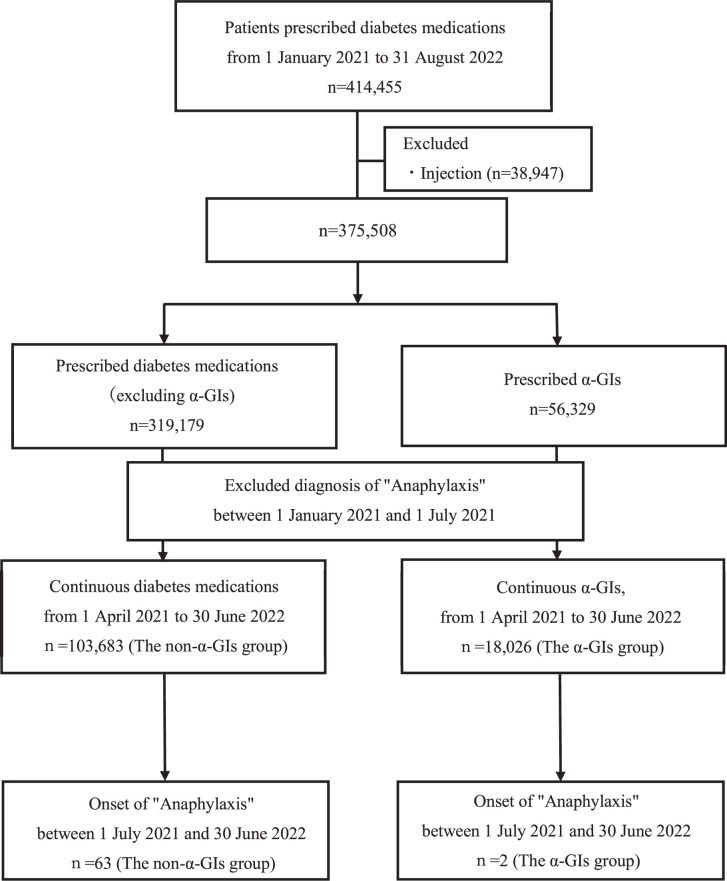
Flowchart of patient eligibility criteria. Patients prescribed diabetes medications between 1 January 2021 and 31 August 2022 (n = 414,455) were identified from the JMDC database. Patients receiving injectable therapies (n = 38,947) were excluded. Patients were categorized as those prescribed diabetes medications excluding α-glucosidase inhibitors (n = 319,179) or those prescribed α-glucosidase inhibitors (n = 56,329). Patients with a diagnosis of anaphylaxis between 1 January 2021 and 1 July 2021 were excluded. Among the remaining patients, those continuously prescribed diabetes medications between 1 April 2021 and 30 June 2022 were included. Patients were then classified into two groups: the non-α-GIs group (n = 103,683) and the α-GIs group (n = 18,026). The primary outcome was the incidence of anaphylaxis between 1 July 2021 and 30 June 2022, with 63 cases in the non-α-GIs group and 2 cases in the α-GIs group. α-GIs, alpha-glucosidase inhibitors.

To assess whether α-GIs, including Acr, are associated with reduced anaphylaxis in humans, we performed an epidemiological analysis using a large-scale Japanese medical database. Among 414,455 individuals, patients with diabetes were identified and classified into non-α-GI (*n* = 103,683) and α-GI-treated groups (*n* = 18,026), including 3 α-GIs (Acr, Mgl and Vgl) (Extended Data Fig. 6). During the observation period, anaphylaxis occurred in 65 patients (0.05%). Of these, 63 cases (0.06%) were observed in the non-α-GI group, whereas only 2 cases (0.01%) occurred in the α-GI-treated group, indicating a lower incidence of anaphylaxis among patients receiving α-GIs (Supplementary Table 5). Notably, no cases of anaphylaxis were observed among patients treated with Acr alone (*n* = 999). Multivariate logistic regression analysis further supported this association, showing that α-GI treatment was associated with a reduced risk of anaphylaxis (odds ratio (OR) = 0.19, 95% confidence interval (CI) = 0.046–0.77; Table 1). Together, these findings suggest that α-GIs are associated with a reduced incidence of anaphylaxis in humans, consistent with the microbiota-dependent protective effects observed in mice.

**Table 1:** Logistic regression analyses of factors associated with anaphylaxis

	OR (95% CI)	*P* value
α-GIs	0.19 (0.46–0.77)	0.0202^*^
Thiazolidine	–	0.9743
Antimicrobial	–	0.9864
Digestive medicine	2.40 (1.02–5.61)	0.0444^*^
H_2_ blocker	1.13 (0.34–3.68)	0.8428
PPIs	1.72 (1.03–2.88)	0.0375^*^
NSAIDs	0.34 (0.05–2.44)	0.2814
Anticancer drug (internal use)	–	0.9936
Anticancer drug (injection)	0.67 (0.09–4.84)	0.6890
Bronchial asthma	1.49 (0.70–3.16)	0.3003
Antihistamine	1.25 (0.45–3.49)	0.6688
Steroid (internal use)	1.09 (0.33–3.53)	0.8897

PPIs, proton pump inhibitors; NSAIDs, nonsteroidal anti-inflammatory drugs. The OR indicates the odds per single unit increase. The OR is for comparison with onset of anaphylaxis.

^*^Results with two-sided *P* values ≤ 0.05 were considered statistically significant.

## Discussion

Acr is widely prescribed for patients with diabetes mellitus and reduces postprandial glucose levels by competitively inhibiting α-glucosidase and α-amylase in the small intestine^16^. By limiting carbohydrate digestion, Acr increases the delivery of dietary digestible carbohydrates to the colon, where they can serve as MACs and alter gut microbial composition^17,18^. However, the physiological consequences of Acr-induced microbial changes have remained unclear. Here we show that Acr functions as a microbiota modulator that suppresses systemic anaphylaxis by reducing mast cell degranulation. This effect required dietary digestible carbohydrates and was associated with increased abundance of *Parabacteroides* and elevated faecal succinate levels. Notably, succinate administration recapitulated the protective phenotype by attenuating mast cell degranulation, supporting its role as a functional mediator. In addition, dietary sucrose and MD promoted the growth of *P. distasonis* and enhanced succinate production in vitro, providing mechanistic support for carbohydrate-dependent metabolic reprogramming. Consistent with these findings in mice, epidemiological analysis revealed a reduced incidence of anaphylaxis in patients with diabetes treated with α-Gis (Supplementary Fig. 1).

Our results indicate that Acr does not suppress mast cell degranulation through modulation of type 2 immune responses or allergen-specific IgE induction. Previous studies have shown that dietary fibre alters gut microbiota composition and increases SCFA production, which promotes T_reg_ cell induction and reduces allergen-specific IgE levels, thereby attenuating allergic responses^13,14^. Although Acr increased RORγt^+^ T_reg_ cells in the siLP and cLP, OVA-specific IgE levels were paradoxically higher in Acr-treated mice. Because IgE class switching is regulated by cytokines such as IL-4 and IL-13 (refs. ^29,30^), and *Il4* and *Il13* expression remained unchanged, we could not identify a mechanism explaining this increase in antigen-specific IgE. Notably, previous studies have reported inconsistent relationships between IgE levels and allergy severity^31–34^, suggesting that qualitative features of IgE, rather than its abundance, may determine anaphylactic responses. For example, IgE glycosylation, including sialylation, modulates FcεRI binding and anaphylaxis severity, and desialylation attenuates systemic anaphylaxis in mouse models^35,36^. Although IgE glycosylation was not assessed in this study, these findings raise the possibility that Acr influences qualitative properties of IgE, potentially explaining the dissociation between IgE levels and anaphylaxis severity observed here.

Several studies have linked gut microbiota to allergy through mechanisms such as T_reg_ induction and barrier regulation^13,14,24,37,38^; however, how microbial signals regulate mast cell degranulation remains poorly understood. Here we identify succinate, a microbiota-derived metabolite, as a functional mediator that suppresses mast cell activation and systemic anaphylaxis. Although Acr increased luminal succinate levels, we did not detect a corresponding rise in systemic compartments, suggesting that its effects may involve transient exposure, local signalling or indirect neuro-immune pathways. Importantly, both in vitro and oral administration experiments showed that succinate is sufficient to attenuate mast cell degranulation, supporting a succinate–mast cell regulatory axis. Notably, the reduction in serum mMCPT-1 following succinate administration was less pronounced than that observed with Acr treatment, suggesting that succinate represents one component of a broader network of microbiota-derived metabolites. This is consistent with previous reports of synergistic immunomodulatory effects of multiple microbial metabolites^39^ and raises the possibility that locally produced succinate exerts spatially restricted or context-dependent effects that are not fully recapitulated by systemic administration. Together, these observations highlight the multifactorial nature of Acr-mediated immune regulation. Mast cells express the succinate receptor GPR91 but not SCFA-associated receptors such as GPR41, GPR43 or GPR109a (ref. ^40^). Although the succinate–GPR91 axis has been implicated in mast cell function^41^, its role remains context dependent. Succinate has been reported to enhance FcεRIα expression in human mast cells^40^, whereas in vivo administration suppresses anaphylaxis by increasing intracellular cAMP and reducing Ca^2+^ influx^42–44^. As GPR91 couples to multiple G-protein subtypes, downstream signalling may vary depending on tissue context^45^. Our findings suggest that microbiota-derived succinate can suppress mast cell activation under specific metabolic conditions, supporting a context-dependent role of succinate–GPR91 signaling in host immune regulation.

Short-read 16S rRNA gene sequencing identified *Parabacteroides* as the most prominently enriched genus following Acr treatment, and full-length 16S rRNA gene sequencing further resolved this to *P. distasonis* as the dominant species driving this expansion. Although *Bifidobacterium* taxa were also co-enriched, their major fermentation products, acetate and lactate, showed only modest, non-significant increases, and acetate did not suppress anaphylaxis. By contrast, Acr markedly increased luminal succinate levels, and *P. distasonis* possesses the metabolic capacity to produce succinate under relevant carbohydrate conditions. Integrating microbial composition, metabolite profiles and functional assays, *P. distasonis* therefore emerges as the most plausible mechanistic contributor to the Acr-associated succinate axis, whereas co-enriched taxa probably reflect broader ecological shifts rather than direct mediators of protection. However, Acr also enriched other succinate-producing taxa, including members of the *Bacteroides* genus, which are known to generate substantial amounts of succinate^46,47^. Thus, the increased succinate observed under Acr treatment is probably a community-level output of a fermentative network rather than the product of a single species. Together, these findings position *P. distasonis* as a key contributor within a broader microbial consortium, linking carbohydrate-driven ecological shifts to functional metabolite production and host immune regulation.

Beyond succinate, Acr induced coordinated changes in broader fermentation pathways, indicating redistribution of metabolic flux within the gut microbiota. Lactate levels showed a modest, non-significant increase in Acr-treated mice that was abolished by antibiotic treatment, suggesting a microbial origin. Because *P. distasonis* did not produce lactate in vitro, this increase probably reflects the activity of other taxa, such as *Bifidobacterium*. Lactate can serve as a substrate for cross-feeding, and consistent with this, butyrate levels trended upwards alongside expansion of the butyrate-producing genus *Anaerostipes*, suggesting enhanced lactate-to-butyrate conversion. By contrast, succinate accumulation was not accompanied by increased propionate, despite the presence of microorganisms capable of converting succinate to propionate. Consistently, propionate levels were unchanged in vivo, and *P. distasonis* did not enhance propionate production under carbohydrate-supplemented conditions in vitro. Instead, succinate and acetate remained the dominant fermentation products, indicating that Acr-driven metabolic reprogramming preferentially favours succinate accumulation over propionate formation. This pattern probably reflects species- and substrate-specific metabolic routing, as *P. distasonis* preferentially produces succinate and acetate even under carbohydrate-rich conditions. In addition, conversion of succinate to propionate is known to be constrained by environmental factors, including CO_2_ availability and vitamin B_12_-dependent enzymatic steps^28,48,49^, which can further promote succinate accumulation. Collectively, these findings indicate that Acr reshapes gut microbial energy metabolism by directing carbohydrate fermentation towards pathway-specific outcomes, resulting in selective succinate enrichment with potential consequences for host immune regulation.

We found that dietary digestible carbohydrates are required for Acr-induced expansion of *Parabacteroides*, and that sucrose and MD promote the growth of *P. distasonis* in vitro. Consistent with this, genome annotation of *P. distasonis* ATCC8503 (JCM5825T) indicates the presence of carbohydrate-degrading enzymes, including α-glucosidase and sucrase–isomaltase, enabling utilization of dietary carbohydrates such as sucrose and MD^50–52^. These substrates are converted into glucose and metabolized through glycolysis to generate pyruvate, which can subsequently fuel succinate production. Thus, *Parabacteroides* preferentially uses dietary digestible carbohydrates for growth and channels carbon flux towards succinate production, providing a mechanistic basis for the link between carbohydrate availability, microbial metabolism and host immune regulation observed in this study.

B-type cytochromes are key components of anaerobic respiratory pathways, including fumarate respiration mediated by fumarate reductase^28,53–55^. In this study, carbohydrate supplementation enhanced both growth and succinate production in *Parabacteroides* while reducing b-type cytochrome abundance. This pattern suggests a metabolic shift towards fermentation-dominant energy production under carbohydrate-rich conditions. In such environments, ATP can be generated via substrate-level phosphorylation, and reducing equivalents are reoxidized through the formation of fermentation products such as succinate and acetate, reducing reliance on membrane-associated electron transport systems. Consistent with this, carbohydrate availability is known to promote fermentative metabolism in gut bacteria, as exemplified by glycan-responsive regulatory systems in *Bacteroides thetaiotaomicron*^56^. Our findings, therefore, indicate that increased carbohydrate availability drives a transition from respiration-associated metabolism towards fermentation, thereby supporting succinate production and redox balance in *Parabacteroides*. Collectively, these results highlight the metabolic flexibility of anaerobic gut bacteria and provide a mechanistic framework linking carbohydrate availability to fermentation-driven metabolite production and host immune regulation.

In clinical practice, three α-GIs—Acr, Mgl and Vgl—are widely prescribed for patients with diabetes mellitus. Because dietary intake, particularly MACs, strongly influences gut microbial metabolism and immune function, a limitation of our cohort is the lack of detailed dietary information. To mitigate potential confounding, we excluded individuals receiving insulin monotherapy, thereby reducing heterogeneity associated with type I diabetes and poorly controlled type II diabetes. As a result, the analysed cohort was enriched for individuals more likely to follow lifestyle-based management, including standardized dietary and exercise interventions. Within this relatively homogeneous population, α-GI treatment was associated with a reduced incidence of anaphylaxis, and this trend was consistently observed across multiple clinical datasets. Although these findings do not establish causality, they support the translational relevance of our experimental results and suggest that pharmacological modulation of carbohydrate availability may represent a potential strategy for reducing anaphylaxis risk in humans.

This study has several limitations that warrant further investigation. First, although our data show that Acr suppresses systemic anaphylaxis even when administered after allergen sensitization, the precise effector-phase mechanisms linking gut microbial and metabolic changes to mast cell regulation in vivo remain incompletely defined. While our findings strongly support mast cells as key downstream targets, we did not directly delineate how microbial-derived signals are conveyed to mast cells across anatomical compartments. Second, although Acr treatment markedly increased succinate levels in the gut lumen and succinate was sufficient to suppress mast cell activation, we were unable to determine the exact spatial and temporal dynamics of succinate signaling in vivo. Thus, whether luminal succinate acts through direct local exposure, transient systemic signaling or indirect neuro-immune pathways remains unresolved. Finally, while real-world clinical data revealed a reduced incidence of anaphylaxis in patients treated with α-GIs, the lack of detailed dietary, microbiota and metabolite information in these datasets limited direct mechanistic validation in humans. Prospective clinical studies incorporating microbiome and metabolomic analyses will be necessary to strengthen translational relevance.

## Methods

### Mice

SPF BALB/c and C57BL/6J wild-type (WT) mice (female; 3 or 4 weeks old) were purchased from CLEA Japan. GF BALB/c mice (female; 4 weeks old) were purchased from Sankyo Lab Service. Conventional-bred mice were acclimatized by feeding on a 30-kGy γ-irradiated AIN-93G diet (Oriental Yeast) and tap water, and were co-housed for at least 2 weeks before experimental treatments to minimize interindividual and intergroup differences in the gut microbiota. Mice in the Acr-treated group were administered 0.5% (*w*/*v*) Acr (Tokyo Chemical Industry) for 1 week, 3 weeks or 5 weeks, depending on the experiments; otherwise, mice were administered tap water. When FcγRIIb-deficient^57^ mice (provided by T. Takai and H. Nishimura) and IgA-deficient^58^ mice (provided by T. Adachi) (both are C57BL/6J background; female; used for the experiments from 5 weeks old) were used, these mice cohabitated with C57BL/6J WT mice to reduce the effect of gut microbiota differences. GF and *P. distasonis*-mono-associated gnotobiotic mice were maintained in vinyl isolators with access to ad libitum 50 kGy γ-irradiated AIN-93G (Research Diets) and autoclaved tap water. In the experiment using α-GIs, mice were administered 0.125% (*w*/*v*) Mgl or 0.0025% (*w*/*v*) Vgl for 3 weeks. All animal experiments were approved by the local ethics committee at Kitasato University (24-10) and Keio University (A2022-078). Mice were maintained under a 12-h light–dark cycle at 25 ± 2 °C and 50 ± 5% humidity.

### Food antigen-induced diarrhoea and systemic anaphylaxis mouse model

Mice were sensitized with 100 µl of OVA (0.05 mg ml^−1^) (Sigma-Aldrich) and alum (40 mg ml^−1^) (Thermo Fisher) by intraperitoneal injection when the mice were 5 and 6 weeks old. For the oral challenge model, mice were challenged with 50 mg ml^−1^ OVA in D-PBS (−) by oral gavage 9 times^59^. We assessed the incidence and clinical score of diarrhoea according to the established criteria shown in Extended Data Fig. 1b, in which stool consistency was scored as follows: normal (0), soft (1), watery soft (2), paste (3) and diarrhoea (4). For the systemic anaphylaxis model, mice were challenged with 0.25 mg ml^−1^ OVA in D-PBS (−) by intraperitoneal injection (both BALB/c and C57BL/6J mice). Subsequently, we measured the core body temperature every 10 min for 2 h and assessed the clinical score as per the following criteria: 0, normal; 1, stays in a corner and less behaviour; 2, stays in a corner and reacts to sounds; 3, stays in a corner and barely responds to sounds; 4, lying down and not moving; and 5, dead. The core body temperature was measured using IPTT-300 and DAS7007 (Alphax Bio). All mice survived under the conditions used in the study despite the marked hypothermia.

### Antibiotic treatment

Vancomycin, ampicillin and streptomycin were selected based on our previous study^60^, in which this antibiotic combination effectively depleted dominant colonic bacterial populations. Mice were administered 200 µl of antibiotic cocktail (100 mg ml^−1^ of ampicillin and 5 mg ml^−1^ of streptomycin (Nacalai Tesque) and 200 mg ml^−1^ of vancomycin (FUJIFILM Wako Pure Chemical)) by oral gavage 3 times (every 2 days) for 1 week from day 21 onwards (Fig. 3a).

### Cell preparation and cell staining for flow cytometry

Tissue-associated cell preparations were performed as follows. Peritoneal lavage fluids were collected by washing with RPMI1640 with 2% Newborn Calf Serum (NBCS) (Thermo Fisher Scientific) at 120 min after the intraperitoneal challenge; subsequently, the fluid was centrifuged at 500 × *g* for 7 min, the supernatant was removed and cells were resuspended using D-PBS (−) with 2% NBCS solution. MLNs were collected and smashed using a 100-µm mesh. The cell suspension was collected in 15-ml falcon tubes filled with 10 ml of RPMI1640 with 2% NBCS. The suspension was centrifuged at 500 × *g* for 7 min, the supernatant was removed and cells were resuspended in D-PBS (−) with 2% NBCS solution. siLP and cLP cells were collected as follows. After the jejunum and whole colon were collected, these tissues were cut into about 1-cm pieces and washed with cold D-PBS (−) by vortexing to remove mucus. Intestinal epithelial layers were removed and dissociated using an orbital shaker at 37 °C for 30 min in a 1% dithiothreitol and 20 mM EDTA-containing HBSS (−) solution. Other intestinal tissues were dissociated using 5 mg ml^−1^ Liberase and 0.125 mg ml^−1^ DNase I in RPMI1640 solution using an orbital shaker at 37 °C for 40 min. To remove debris and enrich leukocytes, dissociated cell suspensions were centrifuged with 40% and 80% Percoll solutions, and the cells in the middle layer were collected. After centrifugation (500 × *g* for 7 min) and aspiration of the supernatant, cells were resuspended in D-PBS (−) with 2% NBCS.

Isolated mononuclear cells were incubated with 200-fold diluted anti-CD16/32 antibody in 2% NBCS-containing D-PBS (−) to block Fc receptors before isolated cells were stained with fluorochrome-conjugated antibodies. CD117 (2B8), FcεRIα (MAR-1), CD45 (30-F11), CD3ε (145-2C11), B220 (RA3-6B2), CD11b (M1/70), CD63 (NVG-2), CD4 (GK1.5), IgA (C10-3) and 7-AAD and Fixable Viability Stain 780 were used to stain the dead cells. Subsequently, cells were permeabilized and fixed by using the Transcription Factor Buffer Set (BD Pharmingen). Permeabilized cells were intracellularly stained with the following antibodies: Helios (22F6), GATA3 (L50-823), RORγt (Q31-378) and Foxp3 (FJK-16s). Flow cytometry data were acquired using the MACSQuant flow cytometer (Miltenyi Biotec) and FACSCalibur. The acquired data were analysed using FlowLogic Ver. 7.2 (Inivai Technologies).

### Enzyme-linked immunosorbent assay for serum mMCPT-1

Blood was collected from each mouse either before or 120 min after intraperitoneal challenge and allowed to clot at room temperature (RT) for 2 h. The blood samples were centrifuged at RT at 5,000 × *g* for 15 min, and the supernatants were collected and frozen at −80 °C. Enzyme-linked immunosorbent assay (ELISA) was performed following the manufacturer’s instructions (MCPT-1 Mouse Uncoated ELISA kit; Thermo Fisher Scientific). Serum OVA-specific IgE titres were determined according to the manufacturer’s instructions using the LEGEND MAX Mouse OVA-Specific IgE ELISA kit (BioLegend). OVA-specific IgA and IgG subclasses were measured by ELISA as follows. High-binding plates were coated with OVA (1 mg ml^−1^ in PBS; 100 µl per well) and incubated overnight. After blocking with 1% BSA in PBS, serially diluted serum samples were applied to the plates. Bound antibodies were detected using HRP-conjugated goat anti-mouse IgA, IgG1, IgG2a or IgG2b antibodies (Bethyl Laboratories). Colour development was achieved using 1-Step TMB ELISA Substrate Solution (Thermo Fisher Scientific), and the reaction was stopped by adding 50 µl of 1.2 M H_2_SO_4_. Absorbance was measured at 450 nm with reference subtraction at 570 nm using a SpectraMax plate reader.

### Reverse transcription and quantitative PCR

MLNs were collected, immersed in RNA-later (Thermo Fisher Scientific) and stored at −80 °C. Total RNA from MLNs was extracted using a PureLink RNA Mini Kit (Thermo Fisher Scientific) as per the manufacturer’s instructions. RNA was reverse transcribed to obtain cDNA using the ReverTra Ace qPCR RT Master Mix with gDNA Remover (TOYOBO). Primers for *P. distasonis* and *Parabacteroides goldsteinii* were designed based on a previous study^61^. Primer sequences targeting RNA polymerase D (used as a housekeeping gene) and the b-type cytochrome gene were designed using the KEGG database and validated by BLAST analysis. RT-qPCR was performed using StepOnePlus (Thermo Fisher Scientific) with THUNDERBIRD SYBR qPCR Mix (TOYOBO). Oligonucleotide primers were purchased from Integrated DNA Technologies. The sequences of all primers are listed in Supplementary Table 3.

### 16S rRNA gene sequence analysis

Bacterial DNA in mice faeces was extracted using the E.Z.N.A. Stool DNA Kit Pathogen Detection protocol (OMEGA). Purification was performed using a magLEAD 12gc nucleic acid extraction instrument (Precision System Science). The V3–V4 regions of the 16S rRNA gene were amplified by PCR using the following primers: forward, 5′-TCGTCGGCAGCGTCAGATGTGTATAAGAGACAGCCTACGGGNGGCWGCAG-3′ and reverse, 5′-GTCTCGTGGGCTCGGAGATGTGTATAAGAGACAGGACTACHVGGGTATCTAATCC-3′. Amplicon DNA samples were purified using AMPure XP beads (Beckman Coulter), and adaptors were added by PCR using a Nextera XT index kit (Illumina). The sequencing was performed using a MiSeq System (Illumina). Sequencing data were analysed using QIIME2 version 2020.11 (ref. ^62^). The Cutadapt plugin in QIIME2 was used to trim the primer regions from the raw sequences. Sequences without primer regions were processed using the DADA2 algorithm^63^ to construct ASV sequences. After a randomized selection of high-quality sequence reads (15,398), these reads were used for taxonomy assignment using the SILVA database (version 138). Acquired DNA sequences were used for sequence alignment using nucleotide BLAST (16S ribosomal RNA gene sequences). The V3–V4 region of the 16S rRNA gene was used for amplicon sequencing, which provides genus-level resolution but has limited capacity for confident species-level assignment.

LEfSe analysis was performed using the R package *lefser* (version 1.16.2) following the standard workflow provided by the package. Statistical significance was assessed using the Wilcoxon signed-rank test with a cut-off *P* value < 0.05, and the linear discriminant analysis (LDA) score threshold was set to >2.0.

### Full-length 16S rRNA gene amplicon sequencing

Full-length 16S rRNA genes were amplified using the Pacific Biosciences (PacBio) barcoded forward primers (5′-Phos-GCATCNNNNNNNNNNAGRGTTYGATYMTGGCTCAG-3′) and 1492Rmod (5′-Phos-GCATCNNNNNNNNNNRGHTACCTTGTTACGACTT-3′). ‘Phos’ indicates a 5′-phosphate modification, and ‘N’ represents a unique barcode sequence for each sample. Subsequent procedures followed the manufacturer’s protocol ‘Amplification of bacterial full-length 16S gene with barcoded primers’ (PacBio). The sequencing library was prepared using the PacBio SMRTbell Prep Kit 3.0 following the manufacturer’s protocol. Sequencing was performed on the PacBio Revio system with the option Full resolution base qual = TRUE. PacBio HiFi reads were generated automatically using SMRT Link software (v13.0) with default parameters. Demultiplexing was performed with Lima (v2.12.0) under the HIFI-ASYMMETRIC preset.

### Full-length 16S rRNA gene amplicon sequence variant analysis

Full-length 16S rRNA gene amplicon sequence variants (FL16s-ASVs) were inferred from demultiplexed HiFi reads using the DADA2 package (v1.30.0) in R (v4.3.3) according to ‘DADA2 for PacBio workflow’^64^ with slight modifications. The reads were subjected to quality filtering and trimming using the filterAndTrim function with the following parameters: minQ=3, minLen=1300, maxLen=1600, maxN=0, rm.phix=FALSE and maxEE=2. FL16s-ASVs were then subjected to a homology search against SBDI Sativa curated 16S GTDB database (v10) using BLASTN (v2.16) with a maximum e-value cut-off of 1 × 10^−10^. Top hits were determined by the highest bitscore.

### Association analysis

Association analysis was performed using Microbiome Multivariable Associations with Linear Models (MaAsLin3)^65^ with the following settings: normalization = NONE, transform = LOG, fixed_effects = “control-Acarboce” (reference = control), random_effects = NULL, min_abundance = 0.1, min_prevalence = 0.1, zero_threshold = 0 and max_significance = 0.1. The coef and p_val_individual values from the MaAsLin3 model abundance results were used.

### CE-TOFMS metabolome analysis

Faecal samples were freeze-dried and broken by shaking at 1,500 rpm for 10 min with 4 3-mm zirconia beads using a Shake Master NEO (Bio Medical Science). Disrupted samples (10 ± 0.5 mg) were homogenized with 500 µl MeOH containing internal standards (20 µM each of methionine sulfone and D-camphor-10-sulfonic acid) and 100 mg of 0.1-mm and 4 3-mm zirconia–silica beads (Biospec Products). After brisk shaking (1,500 rpm for 5 min) using a Shaker Master NEO, 200 µl of Milli-Q water and 500 µl of chloroform were added. Subsequently, the mixture was stirred again. After centrifugation at 4,600 × *g* and 4 °C for 15 min, the supernatant was transferred to a centrifugal filter tube with a 5-kDa cut-off. The filtrate was concentrated by centrifugation at 40 °C and reconstituted with 40 µl of Milli-Q water. Ionic metabolites were analysed using CE-TOFMS in both positive and negative modes. All CE-TOFMS experiments were performed using an Agilent capillary electrophoresis system (Agilent Technologies). The peak annotation and quantification were performed using the MasterHands software^66^.

### In vitro mast cell degranulation assay

Peritoneal exudate cells were cultured in the presence of IL-3 (WEHI-3 cell culture supernatant) to expand mast cells, achieving >95% purity (CD45^+^CD117^+^FcεRIα^+^)^67^. Mast cells were sensitized with 250 ng ml^−1^ anti-DNP IgE at 37 °C for 18 h, followed by treatment with or without 10 mM or 20 mM succinate (Suc) for 30 min. Degranulation was induced by stimulation with 1 μg ml^−1^ DNP-HSA for 1 h. Cells were stained with the following antibodies (BioLegend): FITC-conjugated anti-CD107a (LAMP-1, clone 1D4B, catalogue number 121606), PE-conjugated anti-CD117 (c-Kit, clone 2B8, catalogue number 105807), APC-conjugated anti-FcεRIα (clone MAR-1, catalogue number 134316), APC-Fire-conjugated anti-CD45 (clone 30-F11, catalogue number 103154) and PE/Cy7-conjugated anti-CD11b (clone M1/70, catalogue number 101216). Degranulated mast cells were gated on live singlets and identified as CD45^+^CD11b^−^CD117^+^ cells. Degranulation was quantified as the percentage of CD107a^+^CD117^+^ double-positive cells.

### Detection of SCFAs by GC–MS

Faecal samples were collected from mice immediately before the intraperitoneal challenge. Faeces and *P. distasonis* culture supernatants were processed as follows. Faeces were weighed, suspended in nine volumes of distilled water (*w*/*v*) and homogenized. Culture supernatants were diluted with nine volumes of distilled water (*v*/*v*). Samples were centrifuged (10,000 × *g*, 4 °C, 5 min), and 200 µl of supernatant was transferred to new tubes. Subsequently, 10 µl of 1 mM 2-ethylbutyric acid (internal standard) and 20 µl of 20% (*w*/*v*) 5-sulfosalicylic acid (for deproteinization) were added and mixed. After deproteinization, 200 µl of supernatant was transferred to new tubes, and 10 µl of 37% HCl was added for acidification. Organic acids were extracted by adding 1 ml of diethyl ether and vortexing for 15 min. The organic phase (500 µl) was transferred to new glass vials, mixed with 50 µl of *N*-*tert*-butyldimethylsilyl-*N*-methyltrifluoracetamide and incubated at room temperature for 24 h for derivatization. Derivatized samples were analysed by gas chromatography–mass spectrometry (GC–MS; JMS-Q1500GC, JEOL) equipped with an HP-5 capillary column (60 m × 0.25 mm × 0.25 µm; Agilent Technologies). Helium (99.9999%) was used as the carrier gas at a flow rate of 1.2 ml min^−1^. The oven temperature was initially set at 50 °C; increased to 70 °C at 10 °C min^−1^, then to 290 °C at 20 °C min^−1^; and held at 290 °C for 3 min. Compound concentrations were quantified by comparing peak areas with those of corresponding standards.

### Culture of bacteria

*P.* *distasonis* JCM 5825T (ATCC 8503) was obtained from JCM RIKEN BRC and was maintained in 20% glycerol solution at −80 °C. The bacteria were streaked onto pre-reduced mGAM agar (Nissui) and incubated for 2 days anaerobically. A single colony was inoculated in mGAM broth (Nissui) (Supplementary Table 4), and the suspension was incubated for 24 h for use in vitro culture experiments and administration to GF mice.

### In vitro bacterial culture experiment

We used AccuDia GAM Semisolid without dextrose (AccuDia, Shimadzu Diagnostics), from which agar was removed by filtration to prepare a liquid medium (hereafter referred to as carbohydrate-free GAM broth). This medium provides peptides, amino acids and other nutrients but contains no added carbohydrates. Thus, while not nutrient free, it is devoid of exogenous carbohydrates and serves as an appropriate ‘no-carbohydrate’ control in the context of our experiments. The 24-h-cultured *P. distasonis* bacterium suspension was centrifuged at 4 °C, 2,000 × *g*, for 10 min, and the supernatant was removed. After being washed with pre-reduced D-PBS (−) twice, the pellet containing *P. distasonis* was resuspended in carbohydrate-free GAM broth. Subsequently, the *P. distasonis* suspension was mixed with each sugar (sucrose or MD)-containing medium (pre-reduced) in a 96-well plate. The plate was sealed in an anaerobic chamber and incubated at 37 °C for 24 h. Optical density at 600 nm (OD_600_) was measured every 1 h for 24 h using SpectraMax (Molecular Devices).

Intracellular NAD^+^ levels were quantified using the EnzyFluo NAD^+^/NADH Assay Kit (BioAssay Systems, catalogue number EFND-100), following the manufacturer’s instructions with minor modifications. *P. distasonis* cells were cultured under anaerobic conditions, collected by centrifugation at 4 °C and washed with ice-cold PBS. Cell pellets were resuspended and lysed in the NAD^+^ extraction buffer supplied with the kit. After incubation, samples were neutralized using the corresponding neutralization buffer and centrifuged, and the supernatants were collected. Each sample was then mixed with the working reagent containing the enzyme mix, fluorescent probe and assay buffer. Fluorescence was measured at *λ* = 530/585 nm using a microplate reader at time zero and after a 30-min incubation at RT. NAD^+^ concentrations were determined based on a standard curve generated using serial dilutions of the provided NAD^+^ standard.

### Raman spectroscopic analysis of *P. distasonis*

*P. distasonis* cultures were centrifuged and washed with D-PBS (−) to remove residual supernatant and culture medium. The resulting cell pellets were mounted under a cover glass and placed on the stage of a confocal Raman microspectrometer equipped with a 532-nm Nd:YAG laser (Compass 315M, Coherent), an inverted microscope (ECLIPSE Ti, Nikon) and a ×100/1.30 NA oil-immersion objective lens (Plan Fluor, Nikon Corporation)^68^. The lateral and depth spatial resolutions of the system were 0.3 μm and 2.6 μm, respectively. Raman mapping was performed for each sample, with the sample area scanned at a 2-μm pitch, a laser power of 10 mW at the sample, and an exposure time of 1.5 s per point, resulting in 441 spectra collected per sample. All spectra were assembled into a single data matrix and subjected to preprocessing as follows^69^. Wavenumber calibration was performed using the Raman spectrum of indene as a standard. Detector sensitivity was corrected using a halogen lamp reference spectrum. All spectra were then subjected to singular-value decomposition (SVD) for noise reduction before multivariate analysis. Multivariate curve resolution–alternating least squares (MCR-ALS) analysis was applied to deconvolve the spectral data and extract distinct biomolecular components and their relative abundance^69^. The component spectra matrix was initialized using SVD-extracted spectral components supplemented with a reference spectrum of glass, with non-negativity constraints imposed on both the spectral and intensity matrices. An additional *l*_1_-norm regularization (Lasso regression) constraint was applied, with the hyperparameter *λ* = 0.0002 determined by cross-validation. To standardize signal intensities across samples and correct for variations in detection sensitivity and sample amount, the MCR-resolved component intensities were normalized to the intensity of the H_2_O Raman band (broad band cantered at ~3,400 cm^−1^, assigned to OH stretch). These normalized values were used for subsequent quantitative analyses and PCA to compare biomolecular profiles across samples^66^

### Epidemiological verification using clinical data

Epidemiological verification was performed by using the Japan Medical Data Center (JMDC) hospital-based administrative claims database (JMDC Inc., Tokyo, Japan). We compared the incidence of anaphylaxis between patients with type 2 diabetes mellitus administered α-GIs and those not administered α-GIs. In addition, multivariate logistic regression analysis was performed to validate whether α-GIs decrease the risk of anaphylaxis even after adjusting for confounding factors. The details are described below. Because only anonymized data were used, the requirement for written informed consent was waived in accordance with the Ethical Guidelines for Medical and Biological Research Involving Human Subjects in Japan, as approved by the relevant ethics committee. Ethical approval was obtained from the Faculty of Pharmacy at Keio University (approval number 240115-1).

#### Data source

The JMDC hospital-based administrative claims database includes data collected from medical institutions in Japan, comprising claims data (hospitalization and outpatient care), diagnosis procedure combination records, clinical laboratory test results and treatment information from April 2014 to August 2022. As of August 2022, the database included approximately 18 million individuals. Diagnoses are coded according to the International Classification of Diseases, Tenth Revision (ICD-10), and drug information is recorded using the World Health Organization Anatomical Therapeutic Chemical (ATC) classification system.

#### Study design and population

This epidemiological validation was conducted as a retrospective cohort study. The patient selection scheme is shown in Extended Data Fig. 6. Patients who had been continuously prescribed antidiabetic medications for at least 3 months prior to 1 July 2021 (index date) were included. The non-α-GI group comprised patients continuously prescribed antidiabetic medications excluding α-GIs, whereas the α-GI group included patients continuously prescribed α-GIs. Exclusion criteria were as follows: (1) patients with a diagnosis of anaphylaxis recorded between 1 January 2021, and 1 July 2021 and (2) patients treated exclusively with insulin (pen or vial) or GLP-1 receptor agonist injections. The incidence of anaphylaxis was evaluated in both groups during the observation period from 1 July 2021 to 30 June 30 2022

#### Outcome

The endpoint in this study was the proportion of anaphylaxis in the non-α-GI and α-GI groups during the observation period. The incidence of anaphylaxis was identified by the ICD-10 code (Supplementary Table 6).

#### Data collection

Baseline characteristics were collected at the index date, including age, sex, medical history, database registration date, diagnosis of anaphylaxis and the date of treatment initiation for anaphylaxis. Treatment-related information was also collected, including prescription history, prescription dates, duration of prescription and route of administration. Medication categories included antidiabetic drugs (Supplementary Table 7), drugs used for symptomatic treatment of allergic conditions (Supplementary Table 8)^70^, drugs potentially affecting the intestinal environment (Supplementary Table 9)^71,72^ and factors associated with drug-induced anaphylaxis (Supplementary Table 10)^70^.

### Statistical analysis

Statistical analyses were performed using GraphPad Prism software (version 10.1.1; GraphPad software). Details of the statistical tests used in various experiments are described in each figure legend and methods. All statistical tests were two-sided analyses. A *P* value of <0.05 was considered statistically significant.

### Reporting summary

Further information on research design is available in the Nature Portfolio Reporting Summary linked to this article.

## Supplementary information

Supplementary Information Supplementary Fig. 1.

Reporting Summary

Supplementary Tables 1–10 Supplementary Table 1: Full-length 16S rRNA gene sequencing. Supplementary Table 2: The formulation of diets. Supplementary Table 3: The list of primers. Supplementary Table 4: The formulation of mGAM medium. Supplementary Table 5: Comparison of characteristics between onset and non-onset of anaphylaxis. Supplementary Table 6: The list of encoded disease names related to anaphylaxis. Supplementary Table 7: The list of encoded disease names related to diabetes medicines. Supplementary Table 8: The list of drug names that decrease the incidence of anaphylaxis. Supplementary Table 9: The list of drug names that affect the intestinal environment. Supplementary Table 10: The list of drug names that influence drug-induced anaphylaxis.

## Source data

Source Data Fig. 1 Statistical source data.

Source Data Fig. 2 Statistical source data.

Source Data Fig. 3 Statistical source data.

Source Data Fig. 4 Statistical source data.

Source Data Fig. 5 Statistical source data.

Source Data Extended Data Fig. 1 Statistical source data.

Source Data Extended Data Fig. 2 Statistical source data.

Source Data Extended Data Fig. 3 Statistical source data.

Source Data Extended Data Fig. 4 Statistical source data.

Source Data Extended Data Fig. 5 Statistical source data.

## Data Availability

Data supporting the findings of this study are available in the article and Supplementary Information. The 16S rRNA gene sequencing data have been deposited in public repositories. Short-read 16S rRNA gene sequencing data are available in the NCBI Sequence Read Archive (https://www.ncbi.nlm.nih.gov/sra) under BioProject IDs PRJNA1370010 and PRJNA1369985. Full-length 16S rRNA gene amplicon sequencing data are available in the DNA Data Bank of Japan (https://www.ddbj.nig.ac.jp/index-e.html) under BioProject ID PRJDB42065. Clinical data were obtained from the JMDC hospital-based administrative claims database (JMDC Inc., Tokyo, Japan; https://www.jmdc.co.jp/en/jmdc-claims-database/). Access to the data is subject to application and data use agreements. Access requests should be directed to JMDC Inc. via their official website. The typical response time is approximately several weeks, depending on the review process. Additional datasets generated during this study are available from the corresponding author upon reasonable request. Source data are provided with this paper.
