# Author Correction: Acarbose redirects gut microbiome utilization of dietary carbohydrates to suppress anaphylaxis in mice

**DOI:** 10.1038/s41564-026-02393-5

**Published:** 2026-05-21

**Authors:** Kyosuke Yakabe, Yukinobu Inoue, Yuki Yanagisawa, Shungo Imai, Shunnosuke Suwa, Masahiro Ando, Yuqing Wu, Rina Kurokawa, Tanakorn Srirat, Takeshi Haneda, Tsuyoshi Miki, Masahiro Ito, Akiyoshi Hirayama, Yosuke Kurashima, Shinji Fukuda, Koji Hase, Wataru Suda, Haruko Takeyama, Satoko Hori, Yun-Gi Kim

**Affiliations:** 1 Research Center for Drug Discovery, Faculty of Pharmacy and Graduate School of Pharmaceutical Sciences, Keio University, Tokyo, Japan; 2 Department of Microbiology, School of Pharmacy, Kitasato University, Tokyo, Japan; 3 Division of Drug Informatics, Faculty of Pharmacy and Graduate School of Pharmaceutical Sciences, Keio University, Tokyo, Japan; 4 Graduate School of Advanced Science and Engineering, Waseda University, Tokyo, Japan; 5 Research Organization for Nano and Life Innovation, Waseda University, Tokyo, Japan; 6 Department of Innovative Medicine, Graduate School of Medicine, Chiba University, Chiba, Japan; 7 Institute for Advanced Academic Research and Research Institute of Disaster Medicine, Chiba University, Chiba, Japan; 8 Chiba University, Synergy Institute for Futuristic Mucosal Vaccine Research and Development (cSIMVa), Chiba, Japan; 9 Department of Medicine, School of Medicine, Chiba University–University of California San Diego Center for Mucosal Immunology, Allergy and Vaccine (CU-UCSD cMAV), San Diego, CA USA; 10 Laboratory for Symbiotic Microbiome Sciences, RIKEN Center for Integrative Medical Sciences, Kanagawa, Japan; 11 Institute for Advanced Biosciences, Keio University, Yamagata, Japan; 12 Transborder Medical Research Center, University of Tsukuba, Ibaraki, Japan; 13 Gut Environmental Design Group, Kanagawa Institute of Industrial Science and Technology, Kanagawa, Japan; 14 Innovative Microbiome Therapy Research Center, Juntendo University Graduate School of Medicine, Tokyo, Japan; 15 Division of Biochemistry, Faculty of Pharmacy and Graduate School of Pharmaceutical Sciences, Keio University, Tokyo, Japan; 16 Research Management Division, The Institute of Fermentation Sciences (IFeS), Faculty of Food and Agricultural Sciences, Fukushima University, Fukushima, Japan; 17 Division of Mucosal Vaccine, International Vaccine Design Center, The Institute of Medical Science, The University of Tokyo (IMSUT), Tokyo, Japan; 18 Human Biology-Microbiome-Quantum Research Center (WPI-Bio2Q), Keio University, Tokyo, Japan; 19 Institute for Advanced Research of Biosystem Dynamics, Graduate School of Advanced Science and Engineering, Waseda Research Institute for Science and Engineering, Waseda University, Tokyo, Japan

**Keywords:** Molecular medicine, Allergy, Microbiome

Correction to: *Nature Microbiology* 10.1038/s41564-026-02350-2, published online 12 May 2026.

In the version of this article initially published, the name of Tanakorn Srirat was listed incorrectly (Srirat Tanakorn) and is now amended in the HTML and PDF versions of the article.

