# Supplementary material for: Acarbose redirects gut microbiome utilization of dietary carbohydrates to suppress anaphylaxis in mice
Source: Nat Microbiol. 2026 May 12;11(6):1598–611. doi: 10.1038/s41564-026-02350-2 (PMC13236591; doi:10.1038/s41564-026-02350-2)
Supplement: Supplementary file 1 — Supplementary Fig. 1. [file 41564_2026_2350_MOESM1_ESM.pdf]

nature microbiology

Supplementary information

https://doi.org/10.1038/s41564-026-02350-2

Acarbose redirects gut microbiome utilization of dietary carbohydrates to suppress anaphylaxis in mice

In the format provided by the authors and unedited

Table of contents

Supplementary Figure 1

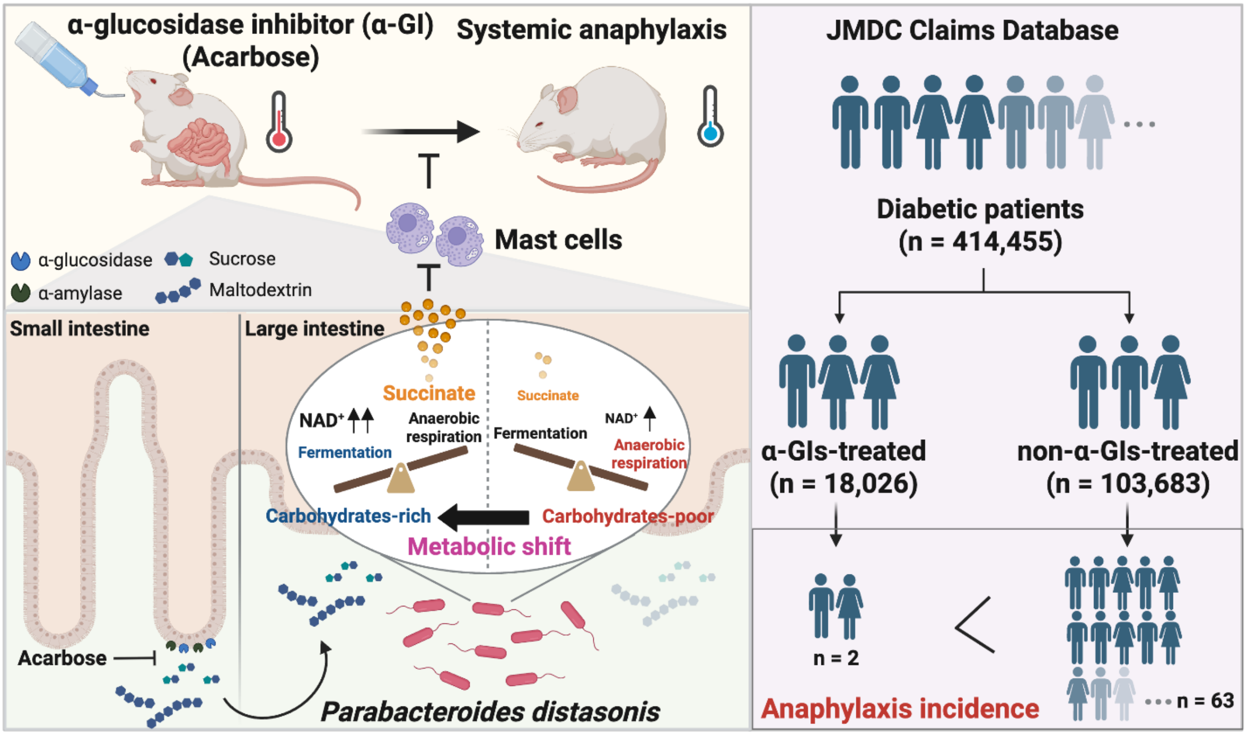

Supplementary Figure 1. Summary of the microbiota–allergy axis revealed in this study. Acarbose redirects dietary carbohydrate metabolism to the colon, promoting the expansion of *Parabacteroides* and a metabolic shift toward succinate-producing fermentation. Microbiota-derived succinate suppresses mast cell degranulation and systemic anaphylaxis. Consistently, the use of α-glucosidase inhibitors is associated with reduced incidence of anaphylaxis in human patients. Illustration created in BioRender; Kim, Y. https://BioRender.com/8rfwjgs (2026).
