# Supplementary material for: Acarbose redirects gut microbiome utilization of dietary carbohydrates to suppress anaphylaxis in mice
Source: Nat Microbiol. 2026 May 12;11(6):1598–611. doi: 10.1038/s41564-026-02350-2 (PMC13236591; doi:10.1038/s41564-026-02350-2)
Supplement: Supplementary file 2 — Reporting Summary [file 41564_2026_2350_MOESM2_ESM.pdf]

nature portfolio

Corresponding author(s): Yun-Gi Kim

Last updated by author(s): 2026/03/22

Reporting Summary

Nature Portfolio wishes to improve the reproducibility of the work that we publish. This form provides structure for consistency and transparency in reporting. For further information on Nature Portfolio policies, see our Editorial Policies and the Editorial Policy Checklist.

Statistics

For all statistical analyses, confirm that the following items are present in the figure legend, table legend, main text, or Methods section.

n/a	Confirmed	
☐	☒	The exact sample size (*n*) for each experimental group/condition, given as a discrete number and unit of measurement
☐	☒	A statement on whether measurements were taken from distinct samples or whether the same sample was measured repeatedly
☐	☒	The statistical test(s) used AND whether they are one- or two-sided<br>*Only common tests should be described solely by name; describe more complex techniques in the Methods section.*
☐	☒	A description of all covariates tested
☐	☒	A description of any assumptions or corrections, such as tests of normality and adjustment for multiple comparisons
☐	☒	A full description of the statistical parameters including central tendency (e.g. means) or other basic estimates (e.g. regression coefficient) AND variation (e.g. standard deviation) or associated estimates of uncertainty (e.g. confidence intervals)
☐	☒	For null hypothesis testing, the test statistic (e.g. *F*, *t*, *r*) with confidence intervals, effect sizes, degrees of freedom and *P* value noted<br>*Give P values as exact values whenever suitable.*
☒	☐	For Bayesian analysis, information on the choice of priors and Markov chain Monte Carlo settings
☒	☐	For hierarchical and complex designs, identification of the appropriate level for tests and full reporting of outcomes
☒	☐	Estimates of effect sizes (e.g. Cohen's *d*, Pearson's *r*), indicating how they were calculated

Our web collection on statistics for biologists contains articles on many of the points above.

Software and code

Policy information about availability of computer code

Data collection	MACSQuant (Miltenyi Biotec), FACSCalibur (BD Bioscience), JMS-Q1500GC (Agilent Technologies), StepOnePlus (Thermo Fisher Scientific), DASHost (Alphax Bio), Miseq (Illumina), CE-TOFMS (Agilent Technologies), Laboratory-built confocal Raman spectrometer (doi: 10.3390/md12052827), SpectraMax (Molecular Devices), PacBio Revio™ system (PacBio)
Data analysis	GraphPad Prism 10 (ver. 10.3.0), RStudio (ver. 1.1.456), FlowLogic (ver. 7.2), FlowJo (ver. 10.7), QIIME2 (ver.2020.11, with DADA2 and SILVA 138 database), Microsoft Excel (ver. 16.99.2), IGOR Pro software (WaveMetrics). Raw data acquired by CE-TOFMS were analyzed using in-house software (MasterHands; doi.org/10.1158/0008-5472.CAN-08-4806), SMRT Link software (v13.0), Lima (v2.12.0), BLASTN (v2.16), MaAsLin3 (https://doi.org/10.1101/2024.12.13.628459).

For manuscripts utilizing custom algorithms or software that are central to the research but not yet described in published literature, software must be made available to editors and reviewers. We strongly encourage code deposition in a community repository (e.g. GitHub). See the Nature Portfolio guidelines for submitting code & software for further information.

Data

Policy information about availability of data

All manuscripts must include a data availability statement. This statement should provide the following information, where applicable:

- Accession codes, unique identifiers, or web links for publicly available datasets
- A description of any restrictions on data availability
- For clinical datasets or third party data, please ensure that the statement adheres to our policy

All data reported in this paper will be shared by the lead contact upon request. The data supporting the findings of this study are available within the paper, its Supplemental Information, and the associated Source Data files. The 16S rRNA gene sequencing data have been deposited in NCBI Sequence Read Archive (SRA) under the BioProject ID PRJNA1370010 and PRJNA1369985. Additional datasets generated during the current study are available from the corresponding author upon reasonable request. The clinical data used in this study were obtained from the JMDC hospital-based administrative claims database (JMDC Inc., Tokyo, Japan) (https://www.jmdc.co.jp/en/jmdc-claims-database/). Any additional information required to reanalyze the data reported in this paper is available from the lead contact upon request. No custom code was used.

Research involving human participants, their data, or biological material

Policy information about studies with human participants or human data. See also policy information about sex, gender (identity/presentation), and sexual orientation and race, ethnicity and racism.

Reporting on sex and gender	Study population consisted of male and female diabetes patients.
Reporting on race, ethnicity, or other socially relevant groupings	The study cohort was extracted from a hospital-based administrative claims database in Japan. Although the majority of the patients are presumed to be Japanese, the database includes individuals who received healthcare services covered by the national insurance system in Japan, which may include non-Japanese residents. No specific inclusion or exclusion criteria were applied based on race or ethnicity.
Population characteristics	To consider the effect of these medications on gut microbiota, patients who were prescribed oral antidiabetic medications were selected. These patients were continuously prescribed diabetic medication at least three months prior to July 1st, 2021. Anaphylaxis events occurring between July 2021 and June 2022 were identified using ICD-10 codes T634, T780, and T782. The onset of anaphylaxis in patients prescribed each medication is shown in Supplemental Table 3.
Recruitment	The design for epidemiological verification was a retrospective cohort study. The populations were collected based on the data registered in JMDC hospital-based administrative claims database. The scheme of recruitment is shown in Supplementary Figure 4.
Ethics oversight	Because only anonymized data were used, the requirement for written informed consent was waived in accordance with the Ethical Guidelines for Medical and Biological Research Involving Human Subjects in Japan. This study was approved by the local ethics committee at Keio University (240115-1), and performed in accordance with ethical guidelines.

Note that full information on the approval of the study protocol must also be provided in the manuscript.

Field-specific reporting

Please select the one below that is the best fit for your research. If you are not sure, read the appropriate sections before making your selection.

☒ Life sciences ☐ Behavioural & social sciences ☐ Ecological, evolutionary & environmental sciences

For a reference copy of the document with all sections, see nature.com/documents/nr-reporting-summary-flat.pdf

Life sciences study design

All studies must disclose on these points even when the disclosure is negative.

Sample size	Sample sizes were not predetermined using statistical methods. Instead, they were chosen based on previous studies and standard practice in the field to ensure sufficient statistical power and reproducibility. The sample sizes used are comparable to those commonly reported in similar experimental systems.
Data exclusions	Based on the quality of sample preparation, flow cytometry data (in Fig.4d and 4e) of one mouse were excluded. No other data were excluded from analysis.
Replication	All experiments were successfully performed at least independently twice or three biologically independent for all results in the study.
Randomization	Animals were randomly assigned to experimental groups where applicable. No specific covariates required control as animals were age- and sex-matched and maintained under the same environmental conditions. Specific gene-deficient mice were co-housed with WT mice in the same cage for at least one week to reduce the effect of the gut microbiota difference before allocating them into experimental groups.
Blinding	The clinical score was blindly evaluated by another investigator by following with the same criteria. All other studies were not strictly blinded

Blinding	because the measurements were based on objective analysis methods.

Reporting for specific materials, systems and methods

We require information from authors about some types of materials, experimental systems and methods used in many studies. Here, indicate whether each material, system or method listed is relevant to your study. If you are not sure if a list item applies to your research, read the appropriate section before selecting a response.

Materials & experimental systems

n/a	Involved in the study
☐	☒ Antibodies
☒	☐ Eukaryotic cell lines
☒	☐ Palaeontology and archaeology
☐	☒ Animals and other organisms
☐	☒ Clinical data
☒	☐ Dual use research of concern
☒	☐ Plants

Methods

n/a	Involved in the study
☒	☐ ChIP-seq
☐	☒ Flow cytometry
☒	☐ MRI-based neuroimaging

Antibodies

Antibodies used

Anti-mouse CD16/32 (BioLegend, Cat# 101320, clone: 97, AB_1574975, Dilution 1: 200)
Fixable Viability Staining 780 (BD Bioscience,Cat# 565388, AB_2869673, Dilution 1: 200)
7-AAD Viability Staining Solution (BioLegend, Cat# 420404, Dilution 1: 200)
BV510 anti-mouse CD45 (BioLegend, Cat# 103138, clone: 30-F11, AB_2563061, Dilution 1: 400)
BV421 anti-mouse GATA3 (BD Bioscience, Cat# 563349, clone: L50-823, AB_2738152, Dilution 1: 200)
FITC anti-mouse FcεRIα (eBioscience, Cat# 11-5898-82, clone: MAR-1, AB_465308, Dilution 1: 100)
APC anti-mouse FcεRIα (BioLegend, Cat# 134316, clone: MAR-1, AB_10640121, Dilution 1: 100)
AF488 anti-mouse/ human Helios (BioLegend, Cat# 137223, clone: 22F6, AB_10661895, Dilution 1: 200)
PerCP-Cy5.5 anti-mouse/ rat Foxp3 (eBioscience, Cat# 45-5773-82, clone: FJK-16s, AB_914351, Dilution 1: 200)
PE-Cy7 anti-mouse CD4 (BioLegend, Cat# 100422, clone: GK1.5, AB_312707, Dilution 1: 200)
APC anti-mouse CD63 (BioLegend, Cat# 143906, clone: NVG-2, AB_2565496, Dilution 1: 200)
FITC anti-mouse CD107a (BioLegend, clone: 1D4B, AB_572007, Dilution 1: 200)
PE anti-mouse CD117 (BioLegend, Cat# 121606, clone: 2B8, AB_313217, Dilution 1: 100)
BV421 anti-mouse CD117 (BioLegend, Cat# 105828, clone: 2B8, AB_11204256, Dilution 1: 100)
APC-Fire anti-mouse CD45 (BioLegend, Cat# 103154, clone: 30-F11, AB_2572116, Dilution 1: 200)
PE-Cy7 anti-mouse CD11b (eBioscience, Cat# 12-0112-82, clone: M1/70, AB_2734869, Dilution 1: 200)
APC-Cy7 anti-mouse TCRβ (BioLegend, Cat# 109220, clone: H57-597, AB_893624, Dilution 1: 200)
FITC anti-mouse IgA (BD Bioscience, Cat# 559354, clone: C10-3, AB_397235, Dilution 1: 200)
PE-Cy7 anti-mouse CD19 (BioLegend, Cat# 115520, clone: 6D5, AB_313655, Dilution 1: 200)
APC anti-mouse CD45R/ B220 (BD Bioscience, Cat# 553092, clone: RA3-6B2, AB_398531, Dilution 1: 200)
FITC anti-mouse CD3ε (BioLegend, Cat# 100306, clone: 145-2C11, AB_312671, Dilution 1: 200)
PE anti-mouse RORγt (BD Bioscience, Cat# 562607, clone: Q31-378, AB_11153137, Dilution 1: 200)
Goat anti-mouse IgA antibody (Bethyl Laboratories Inc., Cat.# A90-103A)
Goat anti-mouse IgA HRP-conjugated antibody (Bethyl Laboratories Inc., Cat.# A90-103P)
Goat anti-mouse IgG antibody (Bethyl Laboratories Inc., Cat.# A90-116A)
Goat anti-mouse IgG1 HRP-conjugated antibody (Bethyl Laboratories Inc., Cat.# A90-105P)
Goat anti-mouse IgG2a HRP-conjugated antibody (Bethyl Laboratories Inc., Cat.# A90-107P)
Goat anti-mouse IgG2b HRP-conjugated antibody (Bethyl Laboratories Inc., Cat.# A90-109P)
Goat anti-mouse IgG2c HRP-conjugated antibody (Bethyl Laboratories Inc., Cat.# A90-136P)

Validation

All antibodies described above are commercially available from BioLegend, BD Biosciences, eBiosciences, and Bethyl Laboratories Inc. Quality validations were performed by each manufacturer. Validation statements are available on the manufacturer's website. Therefore, no extra validation was done in our lab.

Animals and other research organisms

Policy information about studies involving animals; ARRIVE guidelines recommended for reporting animal research, and Sex and Gender in Research

Laboratory animals

Specific pathogen-free (SPF) BALB/c and C57BL/6J wild-type mice (female, 3- or 4-weeks-old) were ordered from CLEA Japan Inc. Germ-free (GF) BALB/c mice (female, 4-weeks-old) were ordered from Sankyo Lab Service Corporation Inc. FcγRIIb-deficient mice (C57BL/6 background; used for the experiments 5-week-old female mice) were kindly given by Drs. Toshiyuki Takai and Hiroyuki Nishimura. IgA-deficient mice (C57BL/6 background; used for the experiments 5-week-old female mice) were kindly given by Dr. Takahiro Adachi. All mice were maintained under 12-hr light/ dark cycle at 23 - 25ºC, and 35 - 45% humidity. GF mice were maintained in the vinyl isolators with feeding 50-kGy-γ-irradiated AIN-93G and autoclaved water.

Wild animals	N/A
Reporting on sex	Only female mice were used in this study.
Field-collected samples	N/A
Ethics oversight	All animal experiments were evaluated and approved by the local ethics committee at Kitasato University (24-10) and Keio University (A2022-078).

Note that full information on the approval of the study protocol must also be provided in the manuscript.

Clinical data

Policy information about clinical studies

All manuscripts should comply with the ICMJE guidelines for publication of clinical research and a completed CONSORT checklist must be included with all submissions.

Clinical trial registration	This is not a clinical trial, but an observational study is included.
Study protocol	This study was approved by the local ethics committee at Keio University (240115-1), and performed in accordance with ethical guidelines.
Data collection	Clinical data were collected from the JMDC hospital-based administrative claims database.
Outcomes	No predefined outcomes were established, as this was an observational study.

Plants

Seed stocks	N/A
Novel plant genotypes	N/A
Authentication	N/A

Flow Cytometry

Plots

Confirm that:

☒ The axis labels state the marker and fluorochrome used (e.g. CD4-FITC).

☒ The axis scales are clearly visible. Include numbers along axes only for bottom left plot of group (a 'group' is an analysis of identical markers).

☒ All plots are contour plots with outliers or pseudocolor plots.

☒ A numerical value for number of cells or percentage (with statistics) is provided.

Methodology

Sample preparation	Lymphocytes in each tissue were collected by following the method described below. Peritoneal fluids were collected by washing with RPMI1640 with 2% Newborn Calf Serum (NBCS) (Thermo Fisher Scientific) at 120 min after the intraperitoneal challenge; subsequently, the fluid was centrifuged at 500 x g for 7 min, the supernatant was removed, and cells were resuspended in D-PBS (-) with 2% NBCS. Mesenteric lymph nodes were harvested and smashed using a 100 µm-mesh. The cell suspension was collected in 15 mL tubes filled with 10 mL of RPMI1640 with 2% NBCS. The suspension was centrifuged at 500 x g for 7 min, the supernatant was removed, and cells were resuspended in D-PBS (-) with 2% NBCS. Small and large intestinal lamina propria cells were collected as follows. After harvesting the jejunum and whole colon, these tissues were cut into about 1 cm pieces and washed with cold D-PBS (-) by vortex. Intestinal epithelial layers were dissociated using an orbital shaker at 37 ºC for 30 min in a 1% dithiothreitol and 20 mM EDTA-containing HBSS (-) solution. Other intestinal tissues were dissociated using 5 mg/mL Liberase™ and 0.125 mg/mL DNase I in RPMI1640 solution using an orbital shaker at 37 ºC for 40 min. To remove debris and enrich leukocytes, dissociated cell suspensions were centrifuged with 40% and 80% Percoll solutions, and the cells in the middle layer were collected. After centrifugation (500 x g for 7 min) and aspiration of the supernatant, cells were resuspended in D-PBS (-) with 2% NBCS.
Instrument	MACSQuant (Miltenyi Biotec) and FACSCalibur (BD Bioscience)

Software	FlowLogic and FlowJo
Cell population abundance	The purity and cell viability were determined by analysis with software. The cell viability (FVS780-negative or 7-AAD-negative) was more than 80% in each analysis.
Gating strategy	Flow cytometry gating strategies were as follows: Single live leukocytes (FSC, SSC, and 7-AAD- or FVS780-negative); mast cells: CD45+, lin (CD3ε, B220, and CD11b)-, CD117+, and FcεRIα +; degranulated mast cells: CD63+. GATA3+ Th cells: CD45+, CD3ε+, CD4+, Helios-, Foxp3-, and GATA3+; RORγt+ pTreg cells: CD45+, CD3ε+, CD4+, Helios-, Foxp3+, and RORγt+.

☒ Tick this box to confirm that a figure exemplifying the gating strategy is provided in the Supplementary Information.
